# Efficacy of a Communication-Priming Intervention on Documented Goals-of-Care Discussions in Hospitalized Patients With Serious Illness: A Randomized Clinical Trial

**DOI:** 10.1001/jamanetworkopen.2022.5088

**Published:** 2022-04-01

**Authors:** Robert Y. Lee, Erin K. Kross, Lois Downey, Sudiptho R. Paul, Joanna Heywood, Elizabeth L. Nielsen, Kelson Okimoto, Lyndia C. Brumback, Susan E. Merel, Ruth A. Engelberg, J. Randall Curtis

**Affiliations:** 1 Cambia Palliative Care Center of Excellence at UW Medicine, University of Washington, Seattle; 2 Division of Pulmonary, Critical Care, and Sleep Medicine, Department of Medicine, Harborview Medical Center, University of Washington, Seattle; 3 University of Washington School of Medicine, Seattle; 4 Department of Family Medicine, University of Washington, Seattle; 5 Department of Biostatistics, University of Washington, Seattle; 6 Division of General Internal Medicine, Department of Medicine, University of Washington, Seattle

## Abstract

**Importance:**

High-quality goals-of-care communication is critical to delivering goal-concordant, patient-centered care to hospitalized patients with chronic life-limiting illness. However, implementation and documentation of goals-of-care discussions remain important shortcomings in many health systems.

**Objective:**

To evaluate the efficacy, feasibility, and acceptability of a patient-facing and clinician-facing communication-priming intervention to promote goals-of-care communication for patients hospitalized with serious illness.

**Design, Setting, and Participants:**

This randomized clinical trial enrolled patients from November 6, 2018, to February 18, 2020. The setting was 2 hospitals in an academic health care system in Seattle, Washington. Participants included hospitalized adults with chronic life-limiting illness, aged 65 years or older and with markers of frailty, or aged 80 years or older. Data analysis was performed from August 2020 to August 2021.

**Intervention:**

Patients were randomized to usual care with baseline questionnaires (control) vs the Jumpstart communication-priming intervention. Patients or surrogates in the intervention group and their clinicians received patient-specific Jumpstart Guides populated with data from questionnaires and the electronic health records (EHRs) that were designed to prompt and guide a goals-of-care discussion.

**Main Outcomes and Measures:**

The primary outcome was EHR documentation of a goals-of-care discussion between randomization and hospital discharge. Additional outcomes included patient-reported or surrogate-reported goals-of-care discussions, patient-reported or surrogate-reported quality of communication, and intervention feasibility and acceptability.

**Results:**

Of 428 eligible patients, this study enrolled 150 patients (35% enrollment rate; mean [SD] age, 59.2 [13.6] years; 66 women [44%]; 132 [88%] by patient consent and 18 [12%] by surrogate consent). Seventy-five patients each were randomized to the intervention and control groups. Compared with the control group, the cumulative incidence of EHR-documented goals-of-care discussions between randomization and hospital discharge was higher in the intervention group (16 of 75 patients [21%] vs 6 of 75 patients [8%]; risk difference, 13% [95% CI, 2%-24%]; risk ratio, 2.67 [95% CI, 1.10-6.44]; *P* = .04). Patient-reported or surrogate-reported goals-of-care discussions did not differ significantly between groups (30 of 66 patients [45%] vs 36 of 66 patients [55%]), although the intrarater consistency of patient and surrogate reports was poor. Patient-rated or surrogate-rated quality of communication did not differ significantly between groups. The intervention was feasible and acceptable to patients, surrogates, and clinicians.

**Conclusions and Relevance:**

In this randomized clinical trial, a patient-facing and clinician-facing communication priming intervention for seriously ill, hospitalized patients promoted EHR-documented goals-of-care discussions before discharge with good feasibility and acceptability. Communication-priming interventions should be reexamined in a larger randomized clinical trial to better understand their effectiveness in the inpatient setting.

**Trial Registration:**

ClinicalTrials.gov Identifier: NCT03746392

## Key Points

**Question:**

Among hospitalized adults with serious illness, does a patient-specific communication-priming intervention (Jumpstart) targeting both patients and their inpatient clinicians increase documented goals-of-care discussions compared with usual care?

**Findings:**

In this 2-hospital randomized clinical trial of 150 patients, the Jumpstart intervention resulted in a significant increase in electronic health record–documented goals-of-care discussions between randomization and hospital discharge (8% of patients in the usual care group vs 21% in the intervention group). Patient-reported or surrogate-reported goals-of-care discussions did not differ between groups.

**Meaning:**

These findings suggest that a communication-priming intervention may be considered in the inpatient setting to increase goals-of-care discussions.

## Teaser

This randomized clinical trial evaluates the efficacy, feasibility, and acceptability of a patient-facing and clinician-facing communication-priming intervention to promote goals-of-care communication for patients hospitalized with serious illness.

## Introduction

Patients with chronic life-limiting illness who are hospitalized for an acute illness are at high risk for morbidity and mortality. High-quality goals-of-care communication among clinicians, patients, and surrogate decision-makers, and documentation of such discussions, may be important to providing patients with goal-concordant, patient-centered care.^1,2,3,4,5^ Although there is debate about the value of advance care planning for healthy adults,^6,7^ there is more agreement about the importance of goals-of-care discussions with seriously ill patients facing important treatment decisions.^2^ We define goals-of-care discussions as discussion of the “overarching aims of medical care for a patient”^8^ used to inform current or near-future treatment decisions. However, despite the importance of such communication,^9,10,11^ clinicians often fail to engage hospitalized patients with serious illness and their surrogates in goals-of-care discussions.^12,13,14^ This omission may lead to delivery of unwanted treatments.^14,15,16^

To date, there have been few interventions proven to improve goals-of-care communication in hospital settings. The SUPPORT (Study to Understand Prognoses and Preferences for Outcomes and Risks of Treatments) trial^17^ in 1995 described widespread shortcomings in communication of treatment preferences for hospitalized patients, but a multimodal intervention consisting of physician-facing prognostic information and a nurse facilitator did not improve communication. More recently, in a 2010 randomized trial^18^ among hospitalized patients, facilitated advance care planning improved family-assessed quality of end-of-life care, reduced intensity of care at the end of life, and improved psychological outcomes in family members. A 2016 randomized trial^19^ of communication facilitators in intensive care units found that the intervention reduced family members’ symptoms of depression and intensity of end-of-life care. These trials and others support the value of improving goals-of-care communication for hospitalized patients.^1,20^ However, the demand for high-quality goals-of-care communication far exceeds the supply of specialists and facilitators to conduct such discussions.^21,22,23^ Moreover, some patients prefer to have these discussions with the clinicians caring for them, rather than others.^24,25^ There remains a need for effective, scalable interventions promoting high-quality goals-of-care discussions by hospital clinicians for patients with serious illness.

Our group has published 2 randomized clinical trials^26,27^ of a communication-priming intervention for outpatients with chronic illness. Both trials found that the intervention increased patient-reported and clinician-documented goals-of-care discussions and patient-reported quality of communication compared with usual care.^26,27^ However, the efficacy of this intervention for hospitalized patients is not known. In the present study, we conducted a pilot randomized clinical trial evaluating the efficacy, feasibility, and acceptability of a patient-facing and clinician-facing communication-priming intervention that uses patient-specific information to prompt and guide goals-of-care discussions for hospitalized patients with serious illness.

## Methods

### Design

We conducted a randomized clinical trial of a patient-facing and clinician-facing communication-priming intervention designed to promote goals-of-care discussions for seriously ill hospitalized patients. Patients were randomized in a 1:1 ratio using random-sized blocks (range, 2-8) and were stratified by hospital to receive either a patient-facing and clinician-facing communication-priming intervention (Jumpstart Guide) or usual care. The randomization sequence was computer generated, and assignments were concealed using sealed envelopes. This study follows the Consolidated Standards of Reporting Trials (CONSORT) reporting guideline for randomized studies.^28^ The trial protocol and statistical analysis plan are shown in Supplement 1.

### Setting

The study was conducted at 2 teaching hospitals affiliated with UW Medicine, an academic health system in Seattle, Washington: a 529-bed academic quaternary referral center and a 413-bed county-owned tertiary care hospital and level I trauma center. The study was approved by the University of Washington institutional review board.

### Participants

#### Patients or Surrogates and Clinicians

Using an automated daily electronic health record (EHR) report, we systematically screened hospitalized patients admitted to either an acute care service (hospital medicine, family medicine, burn surgery, or hematology-oncology) or an intensive care service (medical, oncology, and surgical intensive care units) for at least 12 hours with any of 3 prespecified routes to eligibility: (1) aged 80 years or older; (2) aged 65 years or older with prespecified markers of frailty; or (3) aged 18 years or older with previously diagnosed chronic life-limiting illness. Frailty was defined by serum albumin less than or equal to 3.0 g/dL (to convert to grams per liter, multiply by 10) within 48 hours of admission^29,30,31,32^ plus documented weight loss of 10 pounds or more over the preceding year.^33,34^ Chronic life-limiting illness was defined by *International Statistical Classification of Diseases and Related Health Problems, Tenth Revision* diagnosis codes in the 24 months before admission for any of the 9 chronic conditions used by the Dartmouth Atlas to study end-of-life care: cancers with poor prognosis (ie, primary malignant neoplasms with poor prognoses and metastatic disease), chronic lung disease, coronary artery disease, congestive heart failure, peripheral vascular disease, moderate-to-severe chronic kidney disease, severe chronic liver disease, diabetes with end-organ damage, and dementia).^35,36^ These conditions are associated with 90% of deaths in the Medicare population.^37^ We excluded patients who had a documented goals-of-care discussion in the EHR between admission and screening, whose discharge was imminent, or who had been transferred to an ineligible clinical service, as assessed by study staff using manual record abstraction. Eligible patients were approached by study staff and were assessed using a brief screening tool for cognitive impairment to assess their capacity to complete in-person, written informed consent.^38^ For patients without capacity, a legal surrogate decision-maker was approached to provide in-person, written informed consent. Eligible clinicians included all physicians, residents, subinterns, and advanced practice practitioners on the patient’s primary inpatient team, as well as any consulting geriatrics and palliative care practitioners.

### Data Collection

All patients or their surrogates were asked to complete 2 questionnaires: a baseline questionnaire at enrollment and a follow-up questionnaire 2 to 3 business days after randomization (eAppendix 1 in Supplement 2). The baseline questionnaire assessed participants’ demographic characteristics (eg, race, ethnicity, education, and marital status), current health status, current treatment focus and orientation, occurrence of prior goals-of-care discussions during this hospitalization, and attitude toward further discussions of goals of care. Race and ethnicity (American Indian or Alaska Native, Asian, Black, mixed race or ethnicity, and Hispanic and non-Hispanic White) were assessed in this study because they have been associated with the occurrence of goals-of-care discussions. The follow-up questionnaire included items measuring participants’ perceptions of the occurrence and quality of goals-of-care communication since hospital admission and, if randomized to the intervention, Jumpstart Guide acceptability. Follow-up questionnaires were always distributed to the same individual who had completed the baseline questionnaire; participants discharged before distribution of the follow-up questionnaire were sent a follow-up questionnaire by mail and contacted by telephone. Clinicians who received a Jumpstart Guide were contacted by email 2 to 3 business days after delivery to complete a questionnaire measuring the acceptability of the Jumpstart Guide.

### Intervention

The Jumpstart Guide intervention was delivered to the patient or surrogate at the enrollment visit. It was designed to prompt and guide a goals-of-care discussion between the patient or surrogate and their treating clinicians. To develop the guide, participants in the intervention group completed an additional set of items in the baseline questionnaire that assessed individual preferences for goals-of-care communication, barriers to and facilitators of such communication, and treatment preferences for cardiopulmonary resuscitation.^39,40,41^ Responses from these items were used to create 2 versions of the guide, one for patients or their surrogates and the other for their clinicians. The guide summarized the patient’s or surrogate’s responses and, on the basis of these responses, provided patient-specific prompts for conducting goals-of-care discussions with the enrolled patient or surrogate (eFigure 1 and eFigure 2 in Supplement 2). These prompts were guided by the VitalTalk communication training model and were adapted for use in the inpatient setting with input from both clinicians and a community advisory board, with the primary changes of enhancing graphics and reducing the number of words on Jumpstart Guides.^38,42^ For patients in the intervention group, (1) a patient-facing or surrogate-facing paper Jumpstart Guide was hand-delivered by study staff to the patient or surrogate; (2) clinician-facing Jumpstart Guides were electronically delivered to all physicians, residents, subinterns, and advanced practice practitioners on the patient’s primary inpatient team, as well as any consulting geriatrics and palliative care practitioners; and, (3) a paper clinician-facing Jumpstart Guide was hand-delivered by study staff to the primary inpatient team’s workroom. Patients or surrogates in the control group did not complete the additional items on the baseline questionnaire, nor were Jumpstart Guides generated or provided to these patients, their surrogates, or their treating clinicians.

### Outcomes

#### Primary Outcome: EHR-Documented Goals-of-Care Discussions

The primary outcome was EHR documentation of a goals-of-care discussion between the dates of randomization and hospital discharge. Goals-of-care discussions were defined as discussion of the “overarching aims of medical care for a patient”^8^ and were measured following conclusion of the trial by retrospective abstraction of all EHR notes written by the patient’s inpatient clinicians (physicians, residents, subinterns, and advance practice practitioners). Documented discussions of new advance care planning documents, referrals to palliative care for goals-of-care discussions, and discussions about hospice referral or comfort care were also considered goals-of-care discussions. We did not consider stand-alone discussions of code status (without other values, goals, or treatments preferences) or citations of past advance care planning documents to be goals-of-care discussions. Abstractors were blinded to all aspects of study implementation, including dates of enrollment and allocation to treatment group. We completed double abstraction for a random selection of 357 of 4642 (8%) clinical notes blinded to prior abstraction. The record abstraction protocol, abstractors’ guide and codebook, and methods for aggregating abstraction results to trial outcomes are presented in eAppendix 2 and eAppendix 3 in Supplement 2.

#### Other Outcomes

Additional outcomes were obtained from follow-up questionnaires completed by patients or surrogates. We collected data on patient-reported or surrogate-reported goals-of-care discussions during the hospitalization at baseline and follow-up using a previously validated single item^27^; patient-reported or surrogate-reported quality of communication measured using the 7-item end-of-life subscale of the Quality of Communication (QOC) survey^26,43,44,45^; acceptability of the Jumpstart Guide to patients, surrogates, and clinicians; and feasibility of the Jumpstart intervention (eAppendix 2 in Supplement 2). Our thresholds for acceptability included that more than 80% of patients and clinicians who remembered receiving a Jumpstart Guide stated that they would probably or definitely recommend its use to other patients. Our threshold for feasibility included that more than 80% of clinicians received the Jumpstart Guides within 1 hour of patient completion of the baseline questionnaire.

### Sample Size

A previous trial^27^ of the Jumpstart intervention in the outpatient setting found a significant increase in documented goals-of-care discussions from 17% to 62% (*P* < .001). To power the trial to detect an increase in documented goals-of-care discussions from 50% in the control group (based on preliminary data)^12^ to 75% in the intervention group with 95% CIs and 80% power, we specified an enrollment target of 75 patients in each group, with the goal of ensuring complete data on 55 patients in each group.

### Statistical Analysis

The effect of the intervention on the primary outcome (EHR-documented goals-of-care discussion between randomization and hospital discharge) and other binary outcomes were evaluated with 2-sided Fisher exact test. The effect of the intervention on patient-reported or surrogate-reported quality of communication was evaluated by fitting a subset of the patient and surrogate QOC questionnaire items as indicators of a unidimensional latent variable, with model loadings and thresholds constrained to equality between the intervention and control groups, and then comparing the estimated means of the latent quality-of-communication variable between groups (eAppendix 2 in Supplement 2). As a sensitivity analysis, we also explored the effect of the intervention on individual QOC questionnaire items and on the summed quality ratings from all 7 QOC questionnaire items using an independent 2-sample *t *test. *P* < .05 (2-sided) was considered significant. All analyses were done with SPSS Statistics software version 27 (IBM Corp) and Mplus statistical software version 8.7 (Muthén & Muthén). Data analysis was performed from August 2020 to August 2021.

## Results

From November 6, 2018, to February 18, 2020, we identified 578 patients who met eligibility criteria; 428 were reached for recruitment, and 150 (mean [SD] age, 59.2 [13.6] years; 66 women [44%]) were enrolled (132 [88%] by patient consent, and 18 [12%] by surrogate consent) (Figure), attaining our targeted enrollment. Seventy-five patients were randomized upon enrollment to each of the intervention and control groups (Table 1). The median (IQR) elapsed time from hospital admission to randomization was 4 (2-6) days, and the median (IQR) total length of hospital stay was 9 (5-15) days (eTable 1 in Supplement 2). The primary EHR-abstracted outcome was complete for all patients. Follow-up questionnaires, completed by the patient or surrogate, were returned for 134 of 150 patients (89%) (Figure); 47 of 150 (31%) of participating patients and surrogates expressed at the time of enrollment that they did not want to discuss their goals of care with their inpatient clinicians. The median (IQR) time from randomization to completion of follow-up questionnaire was 3 (2-7) days, and nonresponse rates did not differ significantly between the control and intervention groups.

**Figure.**
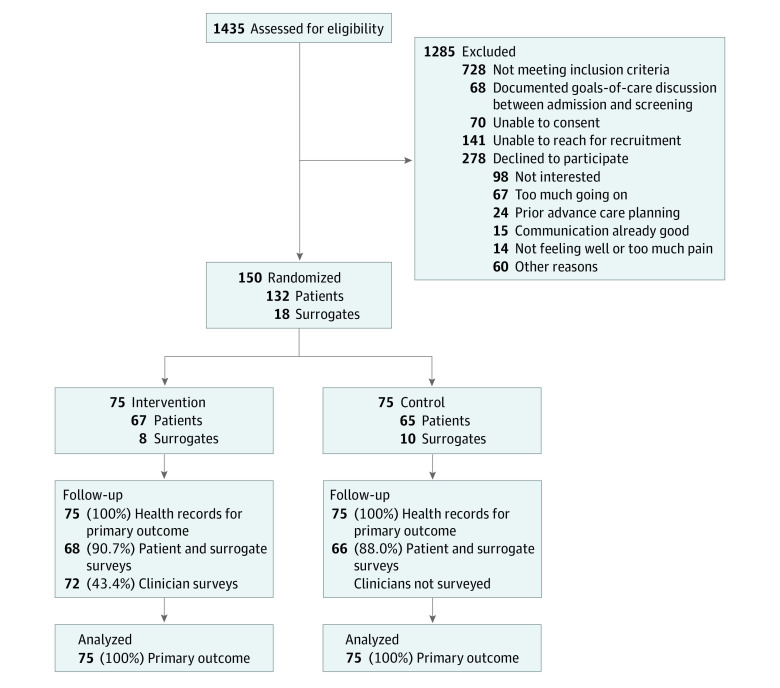
Participant Enrollment Flowchart

**Table 1.** Baseline Characteristics of Enrolled Patients

Characteristic	Patients, No. (%)		
	Total sample (N = 150)	Control group (n = 75)	Intervention group (n = 75)
Respondent			
Patient	132 (88)	65 (87)	67 (89)
Surrogate	18 (12)	10 (13)	8 (11)
Age, median (IQR), y	61 (52-68)	60 (23-92)	62 (20-92)
Sex			
Male	84 (56)	36 (48)	48 (64)
Female	66 (44)	39 (52)	27 (36)
Race or ethnicity^a^			
American Indian or Alaska Native	2 (1)	1 (1)	1 (1)
Asian	2 (1)	2 (3)	0
Black	19 (13)	9 (12)	10 (13)
Mixed race or ethnicity	10 (7)	8 (11)	2 (3)
White			
Hispanic	9 (6)	1 (1)	8 (11)
Non-Hispanic	107 (72)	53 (72)	54 (72)
Educational attainment			
Less than high school diploma	11 (7)	4 (5)	7 (9)
High school diploma or equivalent	26 (17)	18 (24)	8 (11)
Some college	66 (44)	28 (37)	38 (51)
Undergraduate degree	26 (17)	13 (17)	13 (17)
Postcollege education	21 (14)	12 (16)	9 (12)
Marital status^a^			
Single	52 (35)	23 (31)	29 (39)
Currently married	46 (31)	25 (34)	21 (28)
Divorced or widowed	51 (34)	26 (35)	25 (33)
Health status			
Excellent	6 (4)	3 (4)	3 (4)
Very good	6 (4)	4 (5)	2 (3)
Good	24 (16)	13 (17)	11 (15)
Fair	50 (33)	23 (31)	27 (36)
Poor	64 (43)	32 (43)	32 (43)
Chronic life-limiting illnesses, No.			
0	10 (7)	6 (8)	4 (5)
1	64 (43)	33 (44)	31 (41)
2	34 (23)	18 (24)	16 (21)
3	21 (14)	10 (13)	11 (15)
≥4	21 (14)	8 (11)	13 (17)
Eligibility criteria (not mutually exclusive)			
Chronic life-limiting illnesses			
Cancer with poor prognosis	25 (17)	15 (20)	10 (13)
Chronic lung disease	46 (31)	20 (27)	26 (35)
Coronary artery disease	43 (29)	16 (21)	27 (36)
Congestive heart failure	52 (35)	23 (31)	29 (39)
Peripheral vascular disease	21 (14)	11 (15)	10 (13)
Chronic kidney disease, moderate-to-severe	56 (37)	25 (33)	31 (41)
Chronic liver disease, severe	25 (17)	14 (19)	11 (15)
Diabetes with end-organ damage	21 (14)	10 (13)	11 (15)
Dementia	6 (4)	3 (4)	3 (4)
Age ≥65 y with frailty^b^	11 (7)	7 (9)	4 (5)
Age ≥80 y	9 (6)	7 (9)	2 (3)

^a^ Data were missing for 1 patient in the control group.

^b^ Frailty was defined by serum albumin less than or equal to 3.0 g/dL (to convert to grams per liter, multiply by 10) within 48 hours of admission^29,30,31,32^ and documented weight loss of 10 pounds or more over the preceding year.^33,34^

### Effect of Jumpstart Intervention on EHR-Documented Goals-of-Care Discussions

We collected 4642 clinical notes from the index hospitalization for all 150 study participants, of which 109 (2%) were found to contain a documented goals-of-care discussion. Among 357 clinical notes (8%) selected for coabstraction, note-level agreement between abstractors regarding the presence or absence of a documented goals-of-care discussion was 93%.

In retrospective record abstraction of all EHR notes written by the primary inpatient clinician team, the cumulative incidence of EHR-documented goals-of-care discussions from the date of randomization to hospital discharge was higher in the intervention group compared with the control group (16 of 75 patients [21%] vs 6 of 75 patients [8%]; risk difference, 13% [95% CI, 2%- 24%]; risk ratio, 2.67 [95% CI, 1.10-6.44]; *P* = .04) (Table 2). The cumulative incidences of individual EHR documentation domains are shown in eTable 2 in Supplement 2.

**Table 2.** Effect of Intervention on Outcomes

Outcome	Patients, No. (%)		*P* value
	Control group (n = 75)	Intervention group (n = 75)	
Electronic health record–documented goals-of-care discussion	6 (8)	16 (21)	.04^a^
Patient- or surrogate-reported goals-of-care discussion at follow-up			
No. of respondents	66	66	.38^b^
No	28 (42)	34 (52)	
Do not know	2 (3)	2 (3)	
Yes	36 (55)	30 (45)	
Patient- or surrogate-reported quality of communication			
No. of respondents	66	67	.65^c^
Mean (variance)	0.000 (0.398)	0.062 (0.440)	

^a^ *P* value by 2-sided Fisher exact test.

^b^ *P* value by 2-sided Fisher exact test (yes vs no and do not know). In addition to the responses shown, there were 2 additional respondents in the intervention group who skipped this question on the follow-up questionnaire.

^c^ *P* value by a 2-group 5-indicator latent variable model, estimated with weighted least squares. The control group’s estimated mean for the latent quality-of-communication construct was constrained to 0, and the test was for the difference of the intervention group’s mean from that value.

### Patient-Reported and Surrogate-Reported Goals-of-Care Discussions

Among the 132 patients or surrogates who responded to follow-up questionnaires, 36 of 66 patients (55%) in the control group and 30 of 66 patients (45%) in the intervention group reported having had a goals-of-care discussion between hospital admission and follow-up (median [IQR] time after randomization, 3 [2 to 7] days). This difference was not significant (risk difference, 9% [95% CI, –8% to 26%]; risk ratio, 1.20 [95% CI, 0.85 to 1.69]; *P* = .30) (Table 2). Notably, there was poor agreement between patient-reported and surrogate-reported goals-of-care discussions in the follow-up questionnaire and EHR documentation thereof over the same time frame as the questionnaire (ie, from admission to follow-up questionnaire) (54% agreement; κ = 0.10 among 128 respondents who affirmed or denied having had a goals-of-care discussion at follow-up) (eTable 3 in Supplement 2).^46,47,48^ We also observed inconsistencies in patient-reported or surrogate-reported goals-of-care discussions between baseline and follow-up questionnaires: among 57 respondents who reported having had a goals-of-care discussion between admission and the time of the baseline questionnaire, 20 (35%) subsequently reported no goals-of-care discussions between admission and follow-up (eTable 4 in Supplement 2).

### Quality of Communication

Preliminary analysis of the 7 QOC items suggested that the items were not unidimensional. A 2-group model containing the 7 trichotomized items showed substantial misfit of the hypothesized model to the observed data. However, removal of 2 of the indicators (discussing feelings about the possibility that the patient might become more ill and discussing what dying might be like) produced a 5-indicator model with good fit (eFigure 3 in Supplement 2). With the latent variable mean constrained to 0 for the control group, the estimated mean for the intervention group was 0.062, a nonsignificant difference (Table 2). Bivariable analysis of individual questionnaire item responses and of summed quality ratings from all 7 items also showed no significant differences between control and intervention groups (eTable 5 in Supplement 2).

### Acceptability of the Jumpstart Guide

Among the 68 patients and surrogates in the intervention group who responded to follow-up questionnaires, 27 (40%) recalled having used the patient-facing and surrogate-facing Jumpstart Guide to guide a goals-of-care discussion with clinicians. Among these 27 patients and surrogates, 26 (96%) reported that they would probably or definitely recommend its use to other patients.

We distributed 166 follow-up questionnaires to 128 clinicians caring for the 75 patients assigned to the intervention. There were 29 clinicians who received multiple questionnaires for multiple enrolled patients, and some patients had multiple clinicians who returned questionnaires. We received 72 completed questionnaires (43% response rate) from 62 clinicians who had cared for 54 of 75 patients (72%) in the intervention group. Of these 72 questionnaires, 61 (85%) from 52 clinicians for 49 patients indicated that the clinician recalled receiving the Jumpstart Guide. Among the 61 questionnaires from respondents who recalled receiving the Jumpstart Guide, 37 (61%) from 33 clinicians for 31 patients indicated that they used the Jumpstart Guide to facilitate a goals-of-care discussion for the patient, and 54 (89%) from 46 clinicians for 43 patients indicated that they would probably or definitely recommend the Jumpstart Guide to other clinicians.

### Feasibility of the Jumpstart Intervention

Of 428 eligible patients for whom the patient or a surrogate was reached for recruitment, 150 (35%) consented to this study over a 15-month period, meeting our targeted sample size. Patient-facing or surrogate-facing Jumpstart Guides were delivered to all participants in the intervention group within 1 hour of completion of baseline questionnaires. Similarly, clinician-facing Jumpstart Guides were delivered electronically to clinicians from the patient’s primary inpatient service for all patients in the intervention group within 1 hour of completion of baseline questionnaires. The number of clinician-facing Jumpstart Guides delivered per patient ranged from 1 to 5 (median [IQR], 2 [2-3] guides).

## Discussion

In this pilot randomized clinical trial, a patient-facing and clinician-facing communication-priming intervention promoted EHR-documented goals-of-care discussions for hospitalized patients with serious illness. Our findings are consistent with previous trials of the same intervention that took place in the outpatient setting^26,27^ and suggest that the communication behaviors of inpatient clinicians may be modified by a prompting intervention. However, it is important to note that no difference was found in patient-reported or surrogate-reported goals-of-care discussions or quality of communication between the control and intervention groups. In contrast to outpatient clinical encounters, which typically involve a small number of patient-practitioner interactions over a modest time interval, hospitalized patients and their families have a large number of interactions with a multitude of health care practitioners over the course of many days—all while the patient is acutely ill. This milieu is likely to magnify previously described discrepancies between patient-reported, clinician-reported, and EHR-documented goals-of-care discussions in outpatient settings^49^ and may also pose challenges toward measuring quality of communication during hospitalizations. We also observed that many patients and surrogates who reported an inpatient goals-of-care discussion at baseline did not recall or report these same discussions at follow-up a few days later. Although these questionnaire items performed well in a previous outpatient study,^27^ they may be challenging for patients and surrogates to answer in inpatient settings and may not accurately measure the outcome of interest. Overall, our findings suggest promise for the efficacy of communication-priming interventions in promoting EHR-documented goals-of-care discussions for hospitalized patients with serious illness. However, because this was a pilot trial and was limited in size and scope, we believe that additional corroborating evidence is necessary to examine the effectiveness of such interventions in diverse inpatient settings.

Notably, compared with an outpatient study of the same intervention,^27^ the cumulative incidence of documented goals-of-care discussions between randomization and discharge was very low in the control group of this study (8%). Although our study does not examine differences in implementation of goals-of-care discussions between inpatient and outpatient settings, it is notable that 47 of 150 (31%) participating patients and surrogates expressed at the time of enrollment that they did not want to discuss their goals of care with their inpatient clinicians. We suspect that inpatient clinicians may at times feel similarly reticent to explore goals of care with patients. This reticence may arise from the short-term nature of inpatient clinicians’ relationships with patients and families, perceived patient-related and family-related barriers, challenges in prognosticating during acute illness, lack of time, or lack of perceived urgency to discuss goals of care with patients who are not imminently dying.^50,51,52^

Our study also found that implementing a patient-facing and clinician-facing communication-priming intervention is both feasible and acceptable to patients, surrogates, and clinicians in the hospital setting. However, this level of acceptability of the intervention among patients and surrogates who consented to participate may be higher than would be expected in the general population; only 35% of contacted patients or their surrogates consented to participate, suggesting that completion of surveys required for an intervention such as this one in clinical practice may reach only a minority of eligible patients. Redesigning this communication-priming intervention to be clinician-facing only may change its characteristics, but may also enhance scalability and dissemination.^53^

### Limitations

Our study has several important limitations. First, this was a pilot study of modest size. Although the difference we observed between groups in the primary outcome was significant, the results may not generalize to the target population.^54^ Second, although we believe that documentation and communication of goals-of-care discussions across the continuum of care are critical to ensuring the delivery of goal-concordant care,^4,5^ we recognize that EHR-documented goals-of-care discussion during hospitalization is a process measure and that it is not the only criterion by which clinicians’ communication practices should be evaluated. We believe that future studies should assess patients’ and surrogates’ perspectives of inpatient goals-of-care discussions in greater detail, toward the overarching aim of identifying a valid and reliable patient-reported and surrogate-reported measure of goals-of-care communication. Third, our study only enrolled 35% of eligible patients who were contacted, which may introduce nonresponse bias, because patients and surrogates willing to participate in the trial may be more receptive to goals-of-care discussions than others. Fourth, the comparison group in this study received usual care with baseline questionnaires. Although it is possible that the baseline questionnaires or a spillover effect of the interventions primed patients, surrogates, or clinicians to discuss goals-of-care with each other, the low incidence of the primary outcome in the control group makes this unlikely. Fifth, as a 2-hospital study conducted in Washington State, our findings may not generalize to other hospitals or regions.

## Conclusions

In a pilot randomized clinical trial of a patient-facing and clinician-facing communication-priming intervention for hospitalized patients with serious illness, the intervention was efficacious at promoting EHR-documented goals-of-care discussions and was feasible and acceptable to patients, surrogates, and clinicians. Communication-priming interventions should be reexamined in larger randomized trials and with pragmatic approaches to enhance understanding of the effectiveness and implementation of communication-priming interventions in the hospital setting.

## Notes

Supplement 1.
Trial Protocol and Statistical Analysis Plan

Supplement 2. **eAppendix 1.** Participant Questionnaires (Representative Examples) **eFigure 1.** Sample Patient- and Clinician-Facing Jumpstart Guides **eFigure 2.** Matrix of Patient or Surrogate Questionnaire Responses and Clinician Communication Prompts **eAppendix 2.** Supplemental Methods **eAppendix 3.** Chart Abstractors’ Guide and Codebook **eTable 1.** Timing of Enrollment and Randomization, Patient or Surrogate Follow-up Questionnaire, and Length of Hospital Stay **eTable 2.** Cumulative Incidence of Individual Documentation Domains of EHR-Documented Goals-of-Care Discussions Between Randomization and Discharge **eTable 3.** Patient- or Surrogate-Reported vs. EHR-Documented Goals-of-Care Discussions Between Dates of Hospital Admission and Follow-up Survey **eTable 4.** Patient- or Surrogate-Reported Occurrence of a Goals-of-Care Discussion Since Index Admission, at Baseline and Follow-up Questionnaires **eFigure 3.** Two-Group Five-Indicator Latent Quality-of-Communication Model **eTable 5.** Effect of Intervention on Patient or Surrogate Quality of Communication Questionnaire Responses at Follow-up
eReferences

Supplement 3.
Data Sharing Statement
