# Supplementary material for: Efficacy of a Communication-Priming Intervention on Documented Goals-of-Care Discussions in Hospitalized Patients With Serious Illness: A Randomized Clinical Trial
Source: JAMA Netw Open. 2022 Apr 1;5(4):e225088. doi: 10.1001/jamanetworkopen.2022.5088 (PMC8976242; doi:10.1001/jamanetworkopen.2022.5088)
Supplement: Supplement 1. — Trial Protocol and Statistical Analysis Plan [file jamanetwopen-e225088-s001.pdf]

Study Protocol

Title: Program to Improve Communication about Serious Illness – A Pilot Trial

Principal Investigator: J. Randall Curtis, MD, MPH
Co-Principal Investigator: Ruth A. Engelberg, PhD

Cambia Palliative Care Center of Excellence
University of Washington, Seattle WA

<u>Outline</u> **<u>Page</u>**

1) Brief Overview 2
2) Specific Aims 2
3) Research Plan 3
   a) Background and significance 3
   b) Innovation 3
   c) Research design and methods 4
      i) Overview
      ii) Setting
      iii) Patient population
      iv) Study design
      v) Intervention
      vi) Comparison group
      vii) Major outcomes
      viii) Additional outcomes
      ix) Variables describing participants
      x) Quantitative data collection
   d) Aim specific statistical analysis plan 7
   e) Sample size considerations 7
   f) Data management and quality control 8
   g) Protocol modifications 8
   h) Anticipated limitations 8
   i) Anticipated findings 9
4) References 9

1. Brief Overview

Goals-of-care communication represents one of the most important aspects of palliative care, yet remains a major shortcoming in our current healthcare system.[1-5] Electronic health records (EHR) provide a key opportunity to identify many patients who would benefit from goals-of-care discussions, yet few successful interventions have used the EHR to identify patients and promote goals-of-care communication for patients with serious illness. This gap was highlighted in the recent "Research Agenda for Communication between Healthcare Professionals and Patients with Serious Illness".[6]

This pilot study builds on two of our most successful programs, one based in quality improvement and the other in research. First, we implemented a palliative care quality metrics program within a large multi-hospital system using the EHR to identify patients with serious illness and assess the quality of palliative care they received.[7-10] In this program, we are using natural language processing and machine learning (NLP/ML) to identify EHR documentation of goals-of-care discussions.[7] Second, we recently completed a randomized trial that successfully prompted outpatient clinicians to complete goals-of-care discussions with patients with serious illness using our "Jumpstart" intervention. Jumpstart is a "pre-conversation" communication-priming intervention that provides patient-specific information about preferences for communication and care (obtained from patient surveys), as well as tips to improve this communication, in a one-page form initially developed for a randomized trial in the Veterans Affairs system.[11] This form is delivered to patients (to prepare them to talk with clinicians) and clinicians (to provide guidance for goals-of-care communication). In our most recent trial, the intervention increased goals-of-care discussions at a routine outpatient visit from 31% to 75% ($p<0.001$) and increased patient-assessed quality of communication ($p<0.001$).[2,12] However, these interventions relied on manual review of the EHR for identification of eligible patients and did not use the EHR to implement the intervention, which limits scalability and dissemination.

In this pilot study, we use automated EHR information to identify our population of interest: hospitalized patients with serious illness (encompassing multiple acute and chronic illnesses) who do not have EHR documentation of a goals-of-care discussion. We will then conduct a pilot randomized trial of our Jumpstart intervention that provides patient-specific information to clinicians, patients, and family members to prompt and guide goals-of-care discussions. The intervention was informed by Vitaltalk (www.vitaltalk.org) and we strive to accomplish 3 specific aims.

2. Specific Aims:

Specific Aim 1: Conduct a pilot randomized trial to evaluate *feasibility* and *acceptability* of using the EHR to: 1) identify eligible patients; and 2) implement the intervention in the inpatient setting. We will randomize patients to intervention (n=75) or usual care (n=75).

Hypotheses: We will successfully identify and recruit 150 eligible patients. We will implement the intervention with participation by 80% or more of enrolled patients. Patients, family, and clinicians will endorse the intervention as acceptable.

Specific Aim 2: Evaluate the *efficacy* of the intervention for changing processes of care with the primary outcome for this pilot trial being EHR documentation of a goals-of-care discussion. We chose this as the primary outcome to provide support for future funding applications. We will also assess exploratory outcomes to ensure feasibility of outcome assessment including: quality of communication; patients' palliative care needs; patient and family symptoms of anxiety and depression; and patient and family reports of goal-concordant care.

Hypothesis: The intervention will be associated with a significant increase in EHR documentation of goals-of-care discussions and we will successfully collect data for the other outcomes.

Specific Aim 3: Conduct interviews with 30 trial participants from the intervention group (including patients, family members, and clinicians) to identify *barriers and facilitators to the intervention's implementation* in a future trial and into clinical practice.

Anticipated findings: We will identify implementation barriers and facilitators to guide future trials.

3. Research Plan

a. Background and Significance

People near the end of life often receive care they would not choose.[13,14] A recent report from the Institute of Medicine documents these discrepancies in care and identifies advance care planning and goals-of-care discussions as a primary mechanism for addressing them.[13] This type of communication is a focus for improvement for two key reasons: 1) clinicians frequently do not have goals-of-care discussions with their patients until very late in the illness;[1-5] and 2) when these discussions occur, they are associated with improved quality of care and patient- and family-centered outcomes including increased quality of life and fewer intensive treatments at the end of life.[1,15-17]

Goals-of-care discussion should start in the outpatient setting when patients are well enough to participate, in order to inform “in the moment” clinical decisions.[18,19] For hospitalized patients with chronic illness, a key component of high quality care includes goals-of-care discussions conducted early during a hospital stay that build upon prior discussions and identify how patients’ goals of care should inform current care plans.[3,19,20] These early hospital discussions are also supported by the National Quality Forum (NQF).[21] **However, despite their key importance to a large number of patients, these hospital goals-of-care discussions often do not occur.[3,22]**

The recent research agenda in *Annals of Internal Medicine* for serious illness communication highlights the importance of promoting high-quality goals-of-care discussions, as well as the potential opportunity to use the EHR to both identify those patients who would benefit from goals-of-care discussions and to guide clinicians in high-quality discussions.[6] We are conducting a pilot trial to examine the efficacy of such an intervention and facilitate the development and funding of an innovative hybrid effectiveness/implementation trial that evaluates the intervention and its implementation.[23]

b. Innovation

Use of the EHR to identify seriously ill, hospitalized patients without a goals-of-care discussion: We will use our EHR-based quality metrics program to identify hospitalized patients with chronic life-limiting illness or age >80 who do not have EHR documentation of a goals-of-care discussion, thereby targeting a population likely to benefit from the intervention. We proposed using an innovative NLP/ML protocol to identify inpatient and outpatient documentation of goals-of-care discussions; our preliminary data suggested an average accuracy of 90.1% for identifying this documentation, based on a dataset of 722 verified positive goals-of-care notes and 1671 negative notes. However, for this trial we opted to use the gold standard of manual abstraction to identify goals-of-care discussions.

Deliver a bilateral communication-priming intervention for goals-of-care discussions in the hospital setting: The intervention is based on our recently completed randomized trial of the Jumpstart intervention: a patient-specific pre-conversation communication-priming intervention targeting both patients and clinicians by providing each with information obtained from patient- reported surveys that is used to guide a goals-of-care discussion. In an outpatient study of 537 patients, the intervention increased goals-of-care discussions from 31% in the control group to 75% in the intervention ($p<0.001$) and increased patient-assessed quality of communication ($p<0.001$).[2,12] For this pilot trial, we adapted the intervention for hospitalized patients.

Develop an innovative effectiveness/implementation trial that advances implementation science in palliative care: This pilot assesses the feasibility, acceptability, and implementation of its methods and outcomes in the inpatient setting. We will follow this pilot with a novel hybrid effectiveness/implementation trial that would accelerate dissemination of the intervention by allowing us to evaluate implementation strategies and outcomes that may facilitate uptake of the intervention.[23-27] This innovative design offers the opportunity to advance implementation science in palliative care, increasing the utility and fundability of the next grant.

c. Research Design and Methods

i. Overview: We will conduct a pilot randomized trial of an intervention to promote and guide goals-of-care discussions for seriously ill hospitalized patients using an automated method for identifying eligible participants. The trial will assess feasibility and acceptability (Aim 1) as well as efficacy for prompting discussions (Aim 2) and will use qualitative methods to explore barriers and facilitators to implementation and opportunities to improve the intervention (Aim 3).

ii. Setting: We will conduct this study at the two largest hospitals in the UW Medicine system. The University of Washington Medical Center (UWMC) provides tertiary care for the region and has 450 acute care beds and 75 ICU beds. The county safety-net hospital, Harborview Medical Center, is operated by the university and has 350 acute care beds and 94 ICU beds. This facility is the only Level 1 Trauma Center serving five states, and its mission population includes inner city poor, recent immigrants to the US, and persons with HIV/AIDS. These settings offer the advantage of caring for diverse patients while also using a unified EHR incorporating both Cerner and EPIC systems.

iii. Patient population: Eligible patients (aged18 or older) will be identified by ICD-10 codes for at least one of the nine chronic conditions used by the Dartmouth Atlas to study end-of-life care: malignant cancer/leukemia, chronic pulmonary disease, coronary artery disease, congestive heart failure, chronic liver disease, chronic renal disease, dementia, diabetes with end-organ damage, and peripheral vascular disease.[28] These 9 conditions account for 90% of deaths amongst Medicare patients in the US.[29,30] We will also include all hospitalized patients over age 80 as well as patients over age 65 with markers of frailty including albumin level <4.0 within 48 hours of admission[31-34] plus EHR-documented weight loss of ≥10 pounds in the past year.[35,36] Among patients meeting these criteria, we will include only those with no identified documentation of goals-of-care discussions during the current hospitalization, as determined through daily screening of hospitalized patients using methods developed by our palliative care metrics program.[7-10] Patients will be eligible after a 12 hour stay with no maximum stay.

iv. Study design: In this pilot randomized trial, eligible patients will be assigned to intervention or usual care in a 1:1 ratio using variable size blocks and stratifying randomization by hospital.

v. Intervention: The intervention has four components (Figure 1). First, we will use our metrics program to identify seriously ill hospitalized patients. Second, consented patients will complete a survey assessing three domains: a) preferences for discussions about goals of care; b) most important barrier and facilitator for having such discussions; and c) current goals of care. If patients are not able to complete a survey, we will recruit a legal surrogate decision-maker to participate and complete the survey. We will use the protocol from our recent randomized trial to create a “Jumpstart form” to prompt and guide a goals-of-care discussion between the patient and physician team caring for the patient or, if the patient isn’t able, the family member and the physician team (including physicians, nurse practitioners, and physician assistants). Third, we will use our NLP/ML approach to identify goals-of-care discussions, POLST forms, or advance directives in the UW Medicine EHR prior to this admission (inpatient and outpatient) and include this information on the Jumpstart forms. Fourth, we will deliver the Jumpstart form to the primary physician team (all attending and resident physicians, subinterns, and advance practice providers on the primary team caring for the patient) via secure

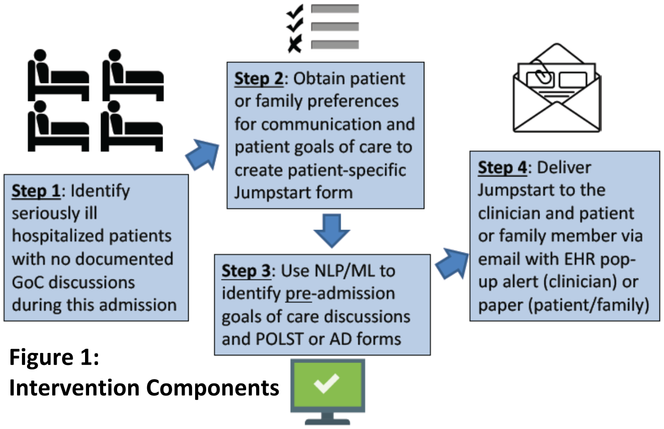

Figure 1: Intervention Components

email with in-person delivery when possible, and we will also provide the patient or family with a patient/family version of the form. The Jumpstart forms will be delivered within 1-2 business days of randomization, as supported by the NQF.[21] The forms provide a distilled version of the patient/family survey responses and, based on the responses, patient-specific suggestions for conducting goals-of-care discussions with this patient or family. The suggestions will be guided by the experience and training of VitalTalk and adapted to the inpatient setting.[37,38] All forms include a link to a 3-minute "just-in-time" training video by VitalTalk on using the form (tailored to clinicians, patients, or family).

vi. Comparison group: The comparison group will receive usual care plus surveys, without steps 3 and 4.

vii. Major outcomes: Aim 1 outcomes assessing the feasibility and acceptability of the intervention will be evaluated with the completion of study activities by those randomized to the intervention. . Aim 2 outcomes will be assessed with patient/family-reported surveys completed by both intervention and comparison groups.

Feasibility and acceptability (Aim 1): Feasibility will be measured with the following: a) survey completion rates with the expectation that successful feasibility will be supported with >80% completion of patient/family surveys; b) receipt of the Jumpstart form with the expectation that >80% of clinicians and patients/families indicate having received the form; and c) use of the form, with the expectation that >80% of clinicians and patients/families will report that they have read the form. We will also add an open-ended question for participants to provide suggestions for improving the intervention.

EHR documentation of goals-of-care discussions (Aim 2): Documentation of goals-of-care discussions will be evaluated using both manual chart review and our NLP/ML methods, with manual chart review using our standard EHR abstraction methods providing the gold standard.[39-41]

Patient or family member report of discussions and quality of discussions (Aim 2): We will survey patients and family members using our previously validated items to assess the occurrence and quality of goals-of-care communication.[11,42-44] Quality of goals-of-care communication will be assessed with the end-of-life communication composite scale (QOC_eol) of the Quality of Communication (QOC) survey. We developed the QOC from qualitative interviews and focus groups with a diverse set of patients, families, and clinicians.[42,43,45] The QOC_eol subscale is based on 4 to 7 items, with item scores potentially ranging from 0 (worst) to 10 (best). We have tested its construct validity through associations with related concepts, such as the number of discussions with the doctor about end-of-life care ($r=0.51$, $p<0.001$), the extent the doctor knows the kinds of treatment wanted if the patient becomes too sick to speak for him/herself ($r=0.39$, $p<0.001$), and a single-item rating of the quality of discussions about end-of-life care ($r=0.43$, $p<0.001$).[42] The QOC_eol scale was responsive to the Jumpstart intervention in our prior trials of this intervention.[2,11,12,16]

viii. Additional outcomes (Aim 2-3): We will collect additional outcomes to help inform the development of the subsequent randomized trial by assessing feasibility of collecting these outcomes in this design.

Symptoms of anxiety and depression: Patient and family member symptoms of anxiety and depression will be assessed with the Hospital Anxiety and Depression Scale (HADS). The HADS is a reliable, valid 14-item, 2-domain (anxiety and depression) tool used to assess symptoms of psychological distress.[46,47] Seven items evaluate anxiety and seven evaluate depression. Each item is scored on a 4-point scale (ranging from 0-3) with scores for each subscale (anxiety and depression) ranging from 0-21. The HADS instrument has been used in over 700 studies with evidence of reliability, validity and responsiveness among patients with acute illness and their family members[48-60] and has become a standard measure for patients and family members after critical illness.[61,62]

Goal-concordant care: Concordance between the care patients want and the care they are receiving will be measured with two questions from SUPPORT.[63] The first question defines patients' priorities for extending life or ensuring comfort: "If you had to make a choice at this time, would you prefer a course of treatment that focuses on extending life as much as possible, even if it means having more pain and discomfort, or would you want a plan of care that focuses on relieving pain and

discomfort as much as possible, even if that means not living as long?" The next question assesses patients' perceptions of current treatment using the same two options.[63] Concordance is defined as a match between preference for care and the type of care currently received, as reported by patients (or families if patients are not able). Although many patients indicate they want *both* quality and life-extending care, this requirement to pick one is a useful way to identify patients' top priority.[64-66] If patients are unable to respond, goals of care are determined by family; this approach mirrors clinical practice. This measure was responsive to the Jumpstart intervention in our recent trial.[2,12] Based on prior studies,[63] we expect only 60% of patients will be receiving care concordant with their goals.[2]

Implementation: We will collect qualitative data on barriers and facilitators for implementation, guided by the Consolidated Framework for Implementation Research, including those related to the intervention, settings (inner and outer), processes, and individuals (see Aim 3 analyses).[26]

ix. Description of participants: For all participants, we will collect age, gender, race/ethnicity, and education (or profession for clinicians). For patients, we will collect comorbidities.[67] For family, we will collect relationship with the patient.

x. Quantitative data collection

Surveys: Surveys will be completed by patients/family at two points in time:1) at enrollment; and 2) at 4-5 business days after randomization. Clinicians will complete surveys at time point 2. Surveys may be completed in-person, online, or by phone, based on respondents' preferences.

Patients and family members: Patients will be surveyed if they are able. Study staff will use a brief six-item screening tool to assess cognitive impairment.[68] If patients are not able to participate, we will identify a legal surrogate decision-maker to consent for both the patient and themselves. The first survey completed at enrollment will include: a) preferences for goals-of-care communication; b) goals of care; c) barriers and facilitators to goals-of-care communication. The second survey, completed 4-5 days later, will include items evaluating occurrence and quality of communication, anxiety and depression (HADS), and goal concordant care. All participants will complete questions about whether they recall using a Jumpstart form and, if yes, was it understandable and useful. In our prior studies, we found some patients in the comparison group who had not received the form mistakenly thought that they received it and, therefore, these are important data to collect.

Clinicians: At patient enrollment, we will collect data on clinicians on the acute care team from hospital records (e.g., age, gender, specialty, level of training) in both arms. After the intervention, a "primary clinician" will be identified for intervention patients (the clinician who did or could have had a goals-of-care discussion) and asked items assessing the intervention: 1) Did he/she complete a goals of care discussions with patient and/or family? 2) If not, what were the reasons for not having had this discussion? 3) Was the Jumpstart used?3) Would he/she recommend the Jumpstart to other clinicians?

EHR: We will use our EHR-based quality metrics program to obtain data about patients from the EHR,[7-10,69] collecting demographics, co-morbidities, and documentation of goals-of-care discussions preceding and during the patient's inpatient stay. In addition, we will conduct a manual chart review to corroborate the documentation of goals-of-care discussions using study staff trained to identify these discussions.[2,39-41]

Qualitative data collection: Aim 3 will use data from 30 semi-structured interviews with patients, family members and clinicians. We will use purposive sampling to ensure a diverse group based on race/ethnicity, age, gender, and, for clinicians, specialty and year of training, Participants will be interviewed by a trained qualitative researcher using an interview guide and interviews will be audio-recorded and transcribed, similar to our prior qualitative research.[70-77]

d. Aim-specific Analyses:

Aim 1: Pilot randomized trial to evaluate the intervention's feasibility and acceptability.

We will assess for successful implementation of the intervention using descriptive statistics to examine the proportion of eligible patients who are enrolled and, among those randomized to the intervention, the proportion of clinicians and patients/families who receive the intervention. We anticipate that 80% of patients randomized to the intervention will receive the intervention and, with our sample size (n=75 intervention patients), we will be able to identify this proportion with 95% confidence intervals of ±7%. We will also examine feasibility of the intervention components as the proportion of enrolled patients for whom each one of the four intervention steps are successfully completed (see Figure 1).

Aim 2: Evaluate the efficacy of the intervention for changing processes of care.

The primary outcome of this trial is the documentation of a goals-of-care discussion in the EHR, which will be assessed with a logistic regression model with adjustment for hospital site and actual confounders. Actual confounders will be patient characteristics (listed above) that change the coefficient for the relationship between intervention-control predictor and the outcome by more than 10%. We will adjust for confounders in this pilot trial in order to maximize the accuracy of the treatment estimate.[78] The intervention's effect on the quality of communication about goals-of-care will be assessed with a composite QOC_eol outcome, collected from patients or family members. The test will use a linear regression model, estimating the coefficient for the outcome regressed on the control/intervention predictor, after adjustment for actual confounders (as above).

We will collect data on the other outcomes to ensure feasibility of outcome assessment with this study design. We will perform descriptive statistics for all outcomes to understand distribution, range, and central tendencies, but we will not report hypothesis testing for these variables in this pilot.[79-81]

Aim 3: Interviews to identify barriers and facilitators for implementation of the intervention.

We will perform a modified grounded theory analysis of transcribed interviews to explore feedback on the intervention, ways to improve the intervention delivery and implementation, and aspects of care not adequately addressed by the intervention.[82-85] Interview guides and analyses will be guided by the Consolidated Framework for Implementation Research to explore factors affecting implementation, within 5 domains: intervention, settings, processes, individuals [26] Qualitative data will be imported to analytic software (Dedoose), where the investigators and coordinator will perform iterative, inductive coding to identify recurrent themes, categories, and relationships among themes and categories. The analysis process will include open coding (identifying major themes and component codes), selective coding (refining themes and codes under each theme), and axial coding (uncovering relationships among themes and codes.)[82,85] To ensure trustworthiness (a qualitative concept similar to reliability in quantitative analysis[85-88]), we will perform a "member check" of the results with participants (n=6) selected for diversity of participant type. We have extensive experience using grounded theory to develop an understanding of palliative care and interventions for improving this care.[71,89-97]

e. Sample size considerations:

Sample size estimation for pilot trials are often determined by requirements associated with feasibility and acceptability assessments, and one should use caution in powering pilot studies based on key outcomes.[79-81] However, we chose to power this pilot study based on a "process of care" outcome – the documentation of goals-of-care discussions – to assess efficacy and facilitate future studies. In our recent outpatient trial, the Jumpstart intervention was associated with a significant increase in documentation of goals-of-care discussions from 17% in the control group to 62% in the intervention group (p<0.001).[2,12] Based on our preliminary data, we estimate that 50% of the control group in this inpatient setting will have documented goals-of-care discussions by the time of death or discharge. This estimate provides for estimates that are maximally conservative, since power increases further

from the 50% mark. We powered this trial to determine if the intervention is able to increase this proportion to 75%, with 95% confidence intervals and power of 80%: this would require a sample size of 55 patients in each group with complete data. We plan to recruit 75 patients in each group to ensure complete data on 55 patients in each arm. This sample size of 75 patients in the intervention group will also provide adequate power to assess feasibility and acceptability.[79-81]

For Aim 3, it is important to achieve theoretical saturation (no new themes emerging).[85,98] We will monitor for saturation, and if saturation is not achieved, we will recruit additional participants.

f. <u>Data management and quality control to achieve scientific rigor</u>

This project requires the creation, maintenance, and analysis of a database that includes a variety of measures from multiple sources. This study, like all studies, depends on the quality of the data and therefore systematic data collection, quality control, and data-management procedures will be implemented: 1) protocols for data collection; 2) rigorous training, certification, and periodic re-training of study staff, with ongoing monitoring of adherence to protocols; 3) regular review of questionnaire response rates, respondent burden,[99] and missing items to identify and correct problems; 4) verification of all data through custom-designed data entry systems; and 5) monthly team meetings and reports to provide feedback to study staff to ensure problems are resolved quickly. To ensure reliability and validity of data, we will use our current methods for training and quality control.[100-104] Staff conducting EHR review will undergo >80 hours of training: instruction on the protocol, guided practice abstraction, and independent abstraction with reconciliation by a trainer. A 10% random sample will be dual-abstracted. We will blind abstractors to randomization status and survey results.

g. <u>Protocol modifications</u>

NLP/ML algorithm: Our NLP/ML algorithm has required ongoing refinement. Hence, for this study we implemented manual abstraction for the purposes of collecting our primary outcome measure, EHR documentation of a goals of care discussion. We are using data gathered via manual abstraction to refine our NLP/ML algorithm. The performance characteristics of this new algorithm are improving over time. A recent version of this algorithm, compared to the standard of manual abstraction, shows a sensitivity of 57%, specificity of 99%, positive predictive value of 53% and negative predictive value of 99%. This produced a positive likelihood ratio of 0.43 and a negative likelihood ratio of 0.71. Although these are good test characteristics, sensitivity and positive predictive values are too low to use this algorithm for outcome adjudication in a randomized trial.

Time to event: Our initial intent was to assess time to goals-of-care discussion as a secondary outcome. However, given the low proportion of events, this analysis was not included.

h. <u>Anticipated limitations</u>

Sample size: The sample size will limit our ability to detect differences between groups for most outcome measures. However, the goals of this pilot study are to assess feasibility and acceptability of the intervention, evaluate for increased documentation of goals-of-care discussions, and develop insights for how to make the intervention more effective. The sample size is adequate for these goals.

Generalizability: This study occurs in a single healthcare system which limits generalizability, but includes two diverse hospitals that use both Cerner and EPIC EHRs, which enhances generalizability. Including additional healthcare systems is not feasible for this pilot.

Scalability of surveys: Study staff will distribute surveys which would not be scalable for broad implementation of the intervention in clinical practice. However, Aim 3 will provide insights into how best to address this limitation for the subsequent hybrid effectiveness-implementation trial.

Quality of communication: Our NLP/ML and manual abstraction approaches identify goals-of-care discussions without assessing their quality. Since our prior trials demonstrated increased patient-

assessed quality with the intervention, this is less of a concern.[2,12] Future NLP/ML advances may permit quality assessments.

i. Anticipated findings

This proposed pilot study is an innovative intervention to improve goals-of-care discussions for seriously ill hospitalized patients and their families. The intervention uses the EHR to identify patients who should have documentation of a goals-of-care discussion but do not, and then prompts and guides this discussion with a bilateral intervention that provides patient-specific support to clinicians, their patients or their family members. The goal of this pilot study is to create the foundation for an innovative effectiveness-implementation trial that would be submitted to the NIH.

j. References

1. Wright AA, Zhang B, Ray A, et al. Associations between end-of-life discussions, patient mental health, medical care near death, and caregiver bereavement adjustment. JAMA 2008;300:1665-73.
2. Fakhri S, Engelberg RA, Downey L, et al. Factors Affecting Patients' Preferences for and Actual Discussions About End-of-Life Care. J Pain Symptom Manage 2016;52:386-94.
3. Heyland DK, Dodek P, You JJ, et al. Validation of quality indicators for end-of-life communication: results of a multicentre survey. CMAJ 2017;189:E980-E9.
4. Silveira MJ, Kim SY, Langa KM. Advance directives and outcomes of surrogate decision making before death. N Engl J Med 2010;362:1211-8.
5. Teno JM, Gruneir A, Schwartz Z, Nanda A, Wetle T. Association between advance directives and quality of end-of-life care: a national study. J Am Geriatr Soc 2007;55:189-94.
6. Tulsky JA, Beach MC, Butow PN, et al. A Research Agenda for Communication Between Health Care Professionals and Patients Living With Serious Illness. JAMA Intern Med 2017;177:1361-6.
7. Curtis JR, Sathitratanacheewin S, Starks H, et al. Using Electronic Health Records for Quality Measurement and Accountability in Care of the Seriously Ill: Opportunities and Challenges. J Palliat Med 2017;epub ahead of print.
8. Lavin K, Davydow DS, Downey L, et al. Effect of Psychiatric Illness on Acute Care Utilization at End of Life From Serious Medical Illness. J Pain Symptom Manage 2017;54:176-85 e1.
9. Hicks K, Downey L, Engelberg RA, et al. Predicator of death in the hospital for patients with chronic serious illness. Journal of Palliative Medicine 2017;epub ahead of print.
10. Sathitratanacheewin S, Engelberg RA, Downey L, et al. Temporal trends between 2010 and 2015 in intensity of care at end-of-life for patients with chronic illness: Influence of age under versus over 65 years. J Pain Symptom Manage 2017;epub ahead of print.
11. Au DH, Udris EM, Engelberg RA, et al. A Randomized Trial to Improve Communication About End-of-Life Care Among Patients With COPD. Chest 2012;141:726-35.
12. Curtis JR, Downey L, Back AL, et al. A patient and clinician communication-priming intervention increases patient-reported goals-of-care discussions between patients with serious illness and clinicians: a randomized trial. 2017;Under review.
13. Institute of Medicine. Improving Quality and Honoring Individual Preferences Near the End of Life. Washington, DC: National Academy Press; 2015.
14. Lorenz K, Lynn J. Morton SC. End-of-Life Care and Outcomes. Summary, Evidence Report/Technology Assessment: Number 110. AHRQ Publication Number 05-E004-1. Agency for Healthcare Research and Quality, 2004. (Accessed February 15, 2012, at http://www.ahrq.gov/clinic/epcsums/eolsum.htm.)
15. Detering KM, Hancock AD, Reade MC, Silvester W. The impact of advance care planning on end of life care in elderly patients: randomised controlled trial. BMJ 2010;340:c1345.
16. Hanson LC, Zimmerman S, Song MK, et al. Effect of the Goals of Care Intervention for Advanced Dementia: A Randomized Clinical Trial. JAMA Intern Med 2017;177:24-31.

17. Torke AM, Callahan CM, Sachs GA, et al. Communication Quality Predicts Psychological Well-Being and Satisfaction in Family Surrogates of Hospitalized Older Adults: An Observational Study. J Gen Intern Med 2017; epub ahead of print.
18. Sudore RL, Fried TR. Redefining the "planning" in advance care planning: preparing for end-of-life decision making. Ann Intern Med 2010;153:256-61.
19. Sudore RL, Lum HD, You JJ, et al. Defining Advance Care Planning for Adults: A Consensus Definition From a Multidisciplinary Delphi Panel. J Pain Symptom Manage 2017;53:821-32 e1.
20. Sinuff T, Dodek P, You JJ, et al. Improving End-of-Life Communication and Decision Making: The Development of a Conceptual Framework and Quality Indicators. J Pain Symptom Manage 2015;49:1070-80.
21. National Quality Forum, Measure 1626: Patients Admitted to ICU who Have Care Preferences Documented within 48 hours (RAND Corporation). 2017. (Accessed January 2, 2018, at https://www.qualityforum.org/News_And_Resources/Endorsement_Summaries/Endorsement_Summaries.aspx.)
22. Heyland DK, Barwich D, Pichora D, et al. Failure to engage hospitalized elderly patients and their families in advance care planning. JAMA Intern Med 2013;173:778-87.
23. Curran GM, Bauer M, Mittman B, Pyne JM, Stetler C. Effectiveness-implementation hybrid designs: combining elements of clinical effectiveness and implementation research to enhance public health impact. Med Care 2012;50:217-26.
24. Peters DH, Adam T, Alonge O, Agyepong IA, Tran N. Implementation research: what it is and how to do it. BMJ 2013;347:f6753.
25. Bauer MS, Damschroder L, Hagedorn H, Smith J, Kilbourne AM. An introduction to implementation science for the non-specialist. BMC Psychol 2015;3:32.
26. Damschroder LJ, Aron DC, Keith RE, Kirsh SR, Alexander JA, Lowery JC. Fostering implementation of health services research findings into practice: a consolidated framework for advancing implementation science. Implement Sci 2009;4:50.
27. Proctor E, Silmere H, Raghavan R, et al. Outcomes for implementation research: conceptual distinctions, measurement challenges, and research agenda. Adm Policy Ment Health 2011;38:65-76.
28. The Dartmouth Atlas of Healthcare. 2016. (Accessed March 25, 2017, at www.dartmouthatlas.org.)
29. Wennberg JE, Bronner K, Skinner JS, Fisher ES, Goodman DC. Inpatient care intensity and patients' ratings of their hospital experiences. Health Aff (Millwood) 2009;28:103-12.
30. Wennberg JE, Fisher ES, Stukel TA, Skinner JS, Sharp SM, Bronner KK. Use of hospitals, physician visits, and hospice care during last six months of life among cohorts loyal to highly respected hospitals in the United States. BMJ 2004;328:607.
31. Goldwasser P, Mittman N, Antignani A, et al. Predictors of mortality in hemodialysis patients. J Am Soc Nephrol 1993;3:1613-22.
32. Foley RN, Parfrey PS, Harnett JD, Kent GM, Murray DC, Barre PE. Hypoalbuminemia, cardiac morbidity, and mortality in end-stage renal disease. J Am Soc Nephrol 1996;7:728-36.
33. Owen WF, Jr., Lew NL, Liu Y, Lowrie EG, Lazarus JM. The urea reduction ratio and serum albumin concentration as predictors of mortality in patients undergoing hemodialysis. N Engl J Med 1993;329:1001-6.
34. Soucie JM, McClellan WM. Early death in dialysis patients: risk factors and impact on incidence and mortality rates. J Am Soc Nephrol 1996;7:2169-75.
35. Zaslavsky AM, Beaulieu ND, Landon BE, Cleary PD. Dimensions of consumer-assessed quality of Medicare managed-care health plans. Medical Care 2000;38:162-74.
36. Fried LP, Tangen CM, Walston J, et al. Frailty in older adults: evidence for a phenotype. J Gerontol A Biol Sci Med Sci 2001;56:M146-56.
37. Back AL, Arnold RM, Baile WF, et al. Efficacy of communication skills training for giving bad news and discussing transitions to palliative care. Arch Intern Med 2007;167:453-60.
38. Back AL, Arnold RM, Tulsky JA, Baile WF, Fryer-Edwards KA. Teaching communication skills to medical oncology fellows. J Clin Oncol 2003;21:2433-6.

39. Curtis JR, Nielsen EL, Treece PD, et al. Effect of a quality-improvement intervention on end-of-life care in the intensive care unit: a randomized trial. Am J Respir Crit Care Med 2011;183:348-55.
40. Curtis JR, Treece PD, Nielsen EL, et al. Integrating palliative and critical care: evaluation of a quality-improvement intervention. Am J Respir Crit Care Med 2008;178:269-75.
41. Curtis JR, Treece PD, Nielsen EL, et al. Randomized Trial of Communication Facilitators to Reduce Family Distress and Intensity of End-of-Life Care. Am J Respir Crit Care Med 2016;193:154-62.
42. Engelberg R, Downey L, Curtis JR. Psychometric characteristics of a quality of communication questionnaire assessing communication about end-of-life care. J Palliat Med 2006;9:1086-98.
43. Curtis JR, Engelberg RA, Nielsen EL, Au DH, Patrick DL. Patient-physician communication about end-of-life care for patients with severe COPD. Eur Respir J 2004;24:200-5.
44. Curtis JR, Patrick DL, Caldwell E, Greenlee H, Collier AC. The quality of patient-clinician communication about end-of-life care: A study of patients with AIDS and their primary care clinicians. AIDS 1999;13:1123-31.
45. Janssen DJ, Curtis JR, Au DH, et al. Patient-clinician communication about end-of-life care for Dutch and US patients with COPD. Eur Respir J 2011;38:268-76.
46. Zigmond AS, Snaith RP. The hospital anxiety and depression scale. Acta Psychiatr Scand 1983;67:361-70.
47. Bjelland I, Dahl AA, Haug TT, Neckelmann D. The validity of the Hospital Anxiety and Depression Scale. An updated literature review. J Psychosom Res 2002;52:69-77.
48. Lautrette A, Darmon M, Megarbane B, et al. A communication strategy and brochure for relatives of patients dying in the ICU. N Engl J Med 2007;356:469-78.
49. Pochard F, Azoulay E, Chevret S, et al. Symptoms of anxiety and depression in family members of intensive care unit patients: Ethical hypothesis regarding decision-making capacity. Crit Care Med 2001;29:1893-7.
50. Azoulay E, Pochard F, Kentish-Barnes N, et al. Risk of post-traumatic stress symptoms in family members of intensive care unit patients. Am J Respir Crit Care Med 2005;171:987-94.
51. Azoulay E, Pochard F, Chevret S, et al. Half the family members of intensive care unit patients do not want to share in the decision-making process: a study in 78 French intensive care units. Crit Care Med 2004;32:1832-8.
52. Azoulay E, Pochard F, Chevret S, et al. Meeting the needs of intensive care unit patient families: a multicenter study. Am J Respir Crit Care Med 2001;163:135-9.
53. Anderson WG, Arnold RM, Angus DC, Bryce CL. Posttraumatic stress and complicated grief in family members of patients in the intensive care unit. J Gen Intern Med 2008;23:1871-6.
54. Anderson WG, Arnold RM, Angus DC, Bryce CL. Passive decision-making preference is associated with anxiety and depression in relatives of patients in the intensive care unit. Journal of critical care 2009;24:249-54.
55. Fumis RR, Deheinzelin D. Family members of critically ill cancer patients: assessing the symptoms of anxiety and depression. Intensive Care Med 2009;35:899-902.
56. Fumis RR, Ranzani OT, Faria PP, Schettino G. Anxiety, depression, and satisfaction in close relatives of patients in an open visiting policy intensive care unit in Brazil. J Crit Care 2015;30:440 e1-6.
57. Fumis RR, Ranzani OT, Martins PS, Schettino G. Emotional disorders in pairs of patients and their family members during and after ICU stay. PLoS One 2015;10:e0115332.
58. Garrouste-Orgeas M, Coquet I, Perier A, et al. Impact of an intensive care unit diary on psychological distress in patients and relatives*. Crit Care Med 2012;40:2033-40.
59. Garrouste-Orgeas M, Philippart F, Timsit JF, et al. Perceptions of a 24-hour visiting policy in the intensive care unit. Crit Care Med 2008;36:30-5.
60. Garrouste-Orgeas M, Willems V, Timsit JF, et al. Opinions of families, staff, and patients about family participation in care in intensive care units. J Crit Care 2010;25:634-40.

61. Needham DM, Sepulveda KA, Dinglas VD, et al. Core Outcome Measures for Clinical Research in Acute Respiratory Failure Survivors. An International Modified Delphi Consensus Study. Am J Respir Crit Care Med 2017;196:1122-30.
62. Connolly B, Hough CL. Coloring by Number? Core Outcome Measures and the Canvas of Intensive Care Unit Survivorship. Am J Respir Crit Care Med 2017;196:1087-9.
63. Teno JM, Fisher ES, Hamel MB, Coppola K, Dawson NV. Medical care inconsistent with patients' treatment goals: association with 1-year Medicare resource use and survival. J Am Geriatr Soc 2002;50:496-500.
64. Coast J, Huynh E, Kinghorn P, Flynn T. Complex Valuation: Applying Ideas from the Complex Intervention Framework to Valuation of a New Measure for End-of-Life Care. Pharmacoeconomics 2016;34:499-508.
65. Finkelstein EA, Bilger M, Flynn TN, Malhotra C. Preferences for end-of-life care among community-dwelling older adults and patients with advanced cancer: A discrete choice experiment. Health Policy 2015;119:1482-9.
66. Flynn TN, Bilger M, Malhotra C, Finkelstein EA. Are Efficient Designs Used in Discrete Choice Experiments Too Difficult for Some Respondents? A Case Study Eliciting Preferences for End-of-Life Care. Pharmacoeconomics 2016;34:273-84.
67. Kajdacsy-Balla Amaral AC, Andrade FM, Moreno R, Artigas A, Cantraine F, Vincent JL. Use of the sequential organ failure assessment score as a severity score. Intensive care medicine 2005;31:243-9.
68. Callahan CM, Unverzagt FW, Hui SL, Perkins AJ, Hendrie HC. Six-item screener to identify cognitive impairment among potential subjects for clinical research. Med Care 2002;40:771-81.
69. Steiner J, Kirkpatrick JN, Heckbert SR, et al. Identification of adults with congenital heart disease of moderate or great complexity from administrative data. Congenital Heart Disease 2017;In press.
70. Back AL, Young JP, McCown E, et al. Abandonment at the end of life from patient, caregiver, nurse, and physician perspectives: loss of continuity and lack of closure. Arch Intern Med 2009;169:474-9.
71. Curtis JR, Engelberg R, Young JP, et al. An approach to understanding the interaction of hope and desire for explicit prognostic information among individuals with severe chronic obstructive pulmonary disease or advanced cancer. J Palliat Med 2008;11:610-20.
72. Carline JD, Curtis JR, Wenrich MD, Shannon SE, Ambrozy DM, Ramsey PG. Physicians' interactions with health care teams and systems in the care of dying patients: Perspectives of dying patients, family members, and health care providers. J Pain Symptom Manage 2003;25:19-28.
73. Curtis JR, Patrick DL, Caldwell E, Collier AC. Why don't patients with AIDS and their clinicians talk about end-of-life care? Barriers to communication for patients with AIDS and their primary care clinicians. Arch Intern Med 2000;160:1690-6.
74. Curtis JR, Wenrich MD, Carline JD, Shannon SE, Ambrozy DM, Ramsey PG. Understanding physicians' skills at providing end-of-life care: Perspectives of patients, families, and health care workers. J Gen Intern Med 2001;16:41-9.
75. Curtis JR, Wenrich MD, Carline JD, Shannon SE, Ambrozy DM, Ramsey PG. Patients' perspectives on physicians' skills at end-of-life care: Differences between patients with COPD, cancer, and AIDS. Chest 2002;122:356-62.
76. Wenrich MD, Curtis JR, Ambrozy DM, et al. Provision of emotional support and personalized care by physicians to patients nearing the end of life. J Pain Symptom Manage 2003;25:236-46.
77. Wenrich MD, Curtis JR, Shannon SE, Carline JD, Ambrozy DM, Ramsey PG. Communicating with dying patients within the spectrum of medical care from terminal diagnosis to death. Arch Intern Med 2001;161:868-74.
78. Hauck WW, Anderson S, Marcus SM. Should we adjust for covariates in nonlinear regression analyses of randomized trials? Controlled Clinical Trials 1998;19:249-56.
79. Kistin C, Silverstein M. Pilot Studies: A Critical but Potentially Misused Component of Interventional Research. JAMA 2015;314:1561-2.

80. Moore CG, Carter RE, Nietert PJ, Stewart PW. Recommendations for planning pilot studies in clinical and translational research. Clin Transl Sci 2011;4:332-7.
81. Leon AC, Davis LL, Kraemer HC. The role and interpretation of pilot studies in clinical research. J Psychiatr Res 2011;45:626-9.
82. Corbin J, Strauss A. Methods of qualitative research. Thousand Oaks, CA: Sage Publications; 1993.
83. Glaser BG. Emerging vs. Forcing: Basics of Grounded Theory Analysis. Mill Valley, CA: Sociology Press; 1992.
84. Glaser BG, Strauss AL. Discovery of Grounded Theory. Chicago: Adline Publishing Company; 1967.
85. Strauss AL, Corbin J. Basics of Qualitative Research: Techniques and Procedures for Developing Grounded Theory. Thousand Oaks: Sage Publications; 1998.
86. Giacomini M, Cook DJ, for the Evidence-Based Medicine Working Group. Qualitative research in health care: Are the results of the study valid? JAMA 2000;284:357-62.
87. Miles MB, Huberman AM. Qualitative Data Analysis: An expanded sourcebook. 2nd ed. Thousand Oaks, CA: Sage Publications; 1994.
88. Stange KC, Miller WL, Crabtree BJ, O'Connor PJ, Zyzanski SJ. Multimethod research: Approaches for integrating qualitative and quantitative research. Journal of General Internal Medicine 1994;9:278-82.
89. Curtis JR, Engelberg RA, Wenrich MD, Au DH. Communication about palliative care for patients with chronic obstructive pulmonary disease. J Palliat Care 2005;21:157-64.
90. Curtis JR, Engelberg RA, Wenrich MD, et al. Studying communication about end-of-life care during the ICU family conference: Development of a framework. J Crit Care 2002;17:147-60.
91. Curtis JR, Engelberg RA, Wenrich MD, Shannon SE, Treece PD, Rubenfeld GD. Missed opportunities during family conferences about end-of-life care in the intensive care unit. Am J Respir Crit Care Med 2005;171:844-9.
92. Engelberg RA, Wenrich MD, Curtis JR. Responding to families' questions about the meaning of physical movements in critically ill patients. J Crit Care 2008;23:565-71.
93. Howell AA, Nielsen EL, Turner AM, Curtis JR, Engelberg RA. Clinician perceptions of the role of an interprofessional communication facilitator to improve family outcomes in the ICU Am J Crit Care 2014;23:380-6.
94. Reinke LF, Engelberg RA, Shannon SE, et al. Transitions regarding palliative and end-of-life care in severe chronic obstructive pulmonary disease or advanced cancer: themes identified by patients, families, and clinicians. J Palliat Med 2008;11:601-9.
95. Reinke LF, Shannon SE, Engelberg RA, Young JP, Curtis JR. Supporting hope and prognostic information: nurses' perspectives on their role when patients have life-limiting prognoses. J Pain Symptom Manage 2010;39:982-92.
96. West HF, Engelberg RA, Wenrich MD, Curtis JR. Expressions of nonabandonment during the intensive care unit family conference. J Palliat Med 2005;8:797-807.
97. White DB, Engelberg RA, Wenrich MD, Lo B, Curtis JR. Prognostication during physician-family discussions about limiting life support in intensive care units. Crit Care Med 2007;35:442-8.
98. Giacomini M, Cook DJ, for the Evidence-Based Medicine Working Group. Qualitative research in health care: What are the results and how do they help me care for my patients? JAMA 2000;284:478-82.
99. Kross EK, Nielsen EL, Curtis JR, Engelberg RA. Survey Burden for Family Members Surveyed About End-of-Life Care in the Intensive Care Unit. Journal of Pain and Symptom Management 2012;44:671-80.
100. Glavan BJ, Engelberg RA, Downey L, Curtis JR. Using the medical record to evaluate the quality of end-of-life care in the intensive care unit. Crit Care Med 2008;36:1138-46.
101. Gries CJ, Engelberg RA, Kross EK, et al. Predictors of symptoms of posttraumatic stress and depression in family members after patient death in the ICU. Chest 2010;137:280-7.
102. Gries CJ, Curtis JR, Wall RJ, Engelberg RA. Family member satisfaction with end-of-life decision making in the ICU. Chest 2008;133:704-12.

103. Gerstel E, Engelberg RA, Koepsell T, Curtis JR. Duration of withdrawal of life support in the intensive care unit and association with family satisfaction. Am J Respir Crit Care Med 2008;178:798-804.

104. Kross EK, Engelberg RA, Gries CJ, Nielsen EL, Zatzick D, Curtis JR. ICU care associated with symptoms of depression and posttraumatic stress disorder among family members of patients who die in the ICU. Chest 2011;139:795-801.
