# Supplementary material for: Efficacy of a Communication-Priming Intervention on Documented Goals-of-Care Discussions in Hospitalized Patients With Serious Illness: A Randomized Clinical Trial
Source: JAMA Netw Open. 2022 Apr 1;5(4):e225088. doi: 10.1001/jamanetworkopen.2022.5088 (PMC8976242; doi:10.1001/jamanetworkopen.2022.5088)
Supplement: Supplement 2. — eAppendix 1. Participant Questionnaires (Representative Examples) eFigure 1. Sample Patient- and Clinician-Facing Jumpstart Guides eFigure 2. Matrix of Patient or Surrogate Questionnaire Responses and Clinician Communication Prompts eAppendix 2. Supplemental Methods eAppendix 3. Chart Abstractors’ Guide and Codebook eTable 1. Timing of Enrollment and Randomization, Patient or Surrogate Follow-up Questionnaire, and Length of Hospital Stay eTable 2. Cumulative Incidence of Individual Documentation Domains of EHR-Documented Goals-of-Care Discussions Between Randomization and Discharge eTable 3. Patient- or Surrogate-Reported vs. EHR-Documented Goals-of-Care Discussions Between Dates of Hospital Admission and Follow-up Survey eTable 4. Patient- or Surrogate-Reported Occurrence of a Goals-of-Care Discussion Since Index Admission, at Baseline and Follow-up Questionnaires eFigure 3. Two-Group Five-Indicator Latent Quality-of-Communication Model eTable 5. Effect of Intervention on Patient or Surrogate Quality of Communication Questionnaire Responses at Follow-up eReferences [file jamanetwopen-e225088-s002.pdf]

Supplemental Online Content

Lee RY, Kross EK, Downey L, et al. Efficacy of a communication-priming intervention on documented goals-of-care discussions in hospitalized patients with serious illness: a randomized clinical trial. *JAMA Netw Open*. 2022;5(4):e225088. doi:10.1001/jamanetworkopen.2022.5088

eAppendix 1. Participant Questionnaires (Representative Examples)
eFigure 1. Sample Patient- and Clinician-Facing Jumpstart Guides
eFigure 2. Matrix of Patient or Surrogate Questionnaire Responses and Clinician Communication Prompts
eAppendix 2. Supplemental Methods
eAppendix 3. Chart Abstractors' Guide and Codebook
eTable 1. Timing of Enrollment and Randomization, Patient or Surrogate Follow-up Questionnaire, and Length of Hospital Stay
eTable 2. Cumulative Incidence of Individual Documentation Domains of EHR-Documented Goals-of-Care Discussions Between Randomization and Discharge
eTable 3. Patient- or Surrogate-Reported vs. EHR-Documented Goals-of-Care Discussions Between Dates of Hospital Admission and Follow-up Survey
eTable 4. Patient- or Surrogate-Reported Occurrence of a Goals-of-Care Discussion Since Index Admission, at Baseline and Follow-up Questionnaires
eFigure 3. Two-Group Five-Indicator Latent Quality-of-Communication Model
eTable 5. Effect of Intervention on Patient or Surrogate Quality of Communication Questionnaire Responses at Follow-up
eReferences

This supplemental material has been provided by the authors to give readers additional information about their work.

© 2022 Lee RY et al. *JAMA Network Open.*

eAppendix 1. Participant Questionnaires (Representative Examples)

(a) Patient baseline questionnaire for control arm

P project to
I improve
C communication about
S serious
I illness
Pilot Study P

Project to Improve Communication about Serious Illness

Thank you for participating in our study to improve communication among doctors, their patients and patients' families.

By improving communication, we hope that we will help patients receive the care they want. Please feel free to skip any questions that you do not want to answer or that you feel do not apply to you. All of your answers are confidential.

If you have questions or need assistance to complete this questionnaire, a member of our research staff would be glad to help you. As you complete the survey, please feel free to add any comments to the margins of the pages or to the space at the end of the survey, or call (206) 744-XXXX to talk with a member of our study team.

Thank you very much. We appreciate your participation.

Please fill in today's date: __________ / __________ /__________
Month Day Year

[CG] Usual Care Group – Baseline Questionnaire for Patient V.7

1

TALKING ABOUT HEALTH CARE

We are interested in knowing about discussions you may have had with your doctors concerning the medical care you would want if you were very sick. These discussions might include whether you would want to receive care such as ICU care, CPR or breathing machines, a nursing home, or hospice care.

1. **<u>In the past, before this hospitalization</u>, have you ever discussed with any of your doctors, in a face-to-face discussion, the kind of medical care you would want if you were too sick to speak for yourself?** *(Please mark one box)*

- 1 ☐ Yes
- 0 ☐ No
- 9 ☐ I don't know

2. **<u>During this current hospital stay</u>, have you discussed with any of the hospital doctors, in a face-to-face discussion, the kind of medical care you would want if you were too sick to speak for yourself?** *(Please mark one box)*

- 1 ☐ Yes
- 0 ☐ No
- 9 ☐ I don't know

3. **Would you like to have a discussion/additional discussions of this type with any of the hospital doctors?** *(Please mark one box)*

- 1 ☐ Yes
- 0 ☐ No
- 9 ☐ I don't know

2

© 2022 Lee RY et al. *JAMA Network Open.*

CHOOSING CARE

We are also interested in the kind of care you prefer and the kind of care you are now receiving.

4. If you had to make a choice at this time, would you prefer a plan of medical care that focuses on <u>extending your life as much as possible</u>, even if it means having more pain and discomfort, or would you want a plan of medical care that focuses on <u>relieving your pain and discomfort as much as possible</u>, even if that means not living as long? *(Please mark one box)*

- 0 ☐ Extending life, even if it means having more pain and discomfort
- 1 ☐ Relieving pain and discomfort as much as possible, even if that means not living as long
- 9 ☐ I'm not sure which I would choose

5. Using those same categories, which of the following best describes the focus of the medical care you are currently receiving? *(Please mark one box)*

- 0 ☐ Extending life, even if it means having more pain and discomfort
- 1 ☐ Relieving pain and discomfort as much as possible, even if that means not living as long
- 9 ☐ I don't know, not sure

YOUR FEELINGS

Please mark the box of the answer that best describes how <u>you</u> have been feeling during the LAST WEEK. Don't think too long over your replies; your immediate answer is best.

6. I feel tense or 'wound up':

- 3 ☐ Most of the time
- 2 ☐ A lot of the time
- 1 ☐ From time to time, occasionally
- 0 ☐ Not at all

7. I still enjoy the things I used to enjoy:

- 0 ☐ Definitely as much
- 1 ☐ Not quite so much
- 2 ☐ Only a little
- 3 ☐ Hardly at all

8. I get a sort of frightened feeling as if something awful is about to happen:

- 3 ☐ Very definitely and quite badly
- 2 ☐ Yes, but not too badly
- 1 ☐ A little, but it doesn't worry me
- 0 ☐ Not at all

9. I can laugh and see the funny side of things:

- 0 ☐ As much as I always could
- 1 ☐ Not quite so much now
- 2 ☐ Definitely not so much now
- 3 ☐ Not at all

© 2022 Lee RY et al. *JAMA Network Open.*

10. Worrying thoughts go through my mind:

- 3 ☐ A great deal of the time
- 2 ☐ A lot of the time
- 1 ☐ From time to time, but not too often
- 0 ☐ Only occasionally

11. I feel cheerful:

- 3 ☐ Not at all
- 2 ☐ Not often
- 1 ☐ Sometimes
- 0 ☐ Most of the time

12. I can sit at ease and feel relaxed:

- 0 ☐ Definitely
- 1 ☐ Usually
- 2 ☐ Not often
- 3 ☐ Not at all

13. I feel as if I am slowed down:

- 3 ☐ Nearly all the time
- 2 ☐ Very often
- 1 ☐ Sometimes
- 0 ☐ Not at all

14. I get a sort of frightened feeling like 'butterflies' in the stomach:

- 0 ☐ Not at all
- 1 ☐ Occasionally
- 2 ☐ Quite Often
- 3 ☐ Very Often

15. I have lost interest in my appearance:

- 3 ☐ Definitely
- 2 ☐ I don't take as much care as I should
- 1 ☐ I may not take quite as much care
- 0 ☐ I take just as much care as ever

16. I feel restless as if I have to be on the move:

- 3 ☐ Very much indeed
- 2 ☐ Quite a lot
- 1 ☐ Not very much
- 0 ☐ Not at all

17. I look forward with enjoyment to things:

- 0 ☐ As much as I ever did
- 1 ☐ Rather less than I used to
- 2 ☐ Definitely less than I used to
- 3 ☐ Hardly at all

© 2022 Lee RY et al. *JAMA Network Open.*

18. I get sudden feelings of panic:

- 3 ☐ Very often indeed
- 2 ☐ Quite often
- 1 ☐ Not very often
- 0 ☐ Not at all

19. I can enjoy a good book or radio or TV program:

- 0 ☐ Often
- 1 ☐ Sometimes
- 2 ☐ Not often
- 3 ☐ Very seldom

20. In the past week, how often have you felt upset or irritable? *(Please mark one box)*

- 1 ☐ Never
- 2 ☐ Rarely
- 3 ☐ Sometimes
- 4 ☐ Often
- 5 ☐ Always

21. In the last week, how often have you felt depressed? *(Please mark one box)*

- 1 ☐ Never
- 2 ☐ Rarely
- 3 ☐ Sometimes
- 4 ☐ Often
- 5 ☐ Constantly

22. In the last week, how often have you felt anxious? *(Please mark one box)*

- 1 ☐ Never
- 2 ☐ Rarely
- 3 ☐ Sometimes
- 4 ☐ Often
- 5 ☐ Constantly

ABOUT YOU

These questions help us describe the people who are participating in the study.

23. What is your <u>current</u> marital status? *(Please mark one box)*

- 0 ☐ Single or never married
- 1 ☐ Married or living with partner
- 2 ☐ Divorced or separated
- 3 ☐ Widowed

24. What is the highest level of schooling that you have completed? *(Please mark one box)*

- 0 ☐ 8th grade or less
- 1 ☐ Some high school
- 2 ☐ High school diploma or GED
- 3 ☐ Some college or trade school
- 4 ☐ 4-year college degree (ex. BA or BS)
- 5 ☐ Some graduate school
- 6 ☐ Graduate degree (ex. MA, MS, PhD, MD)

© 2022 Lee RY et al. *JAMA Network Open.*

25. **In general, would you say your health is:** *(Please mark one box)*

- 0☐ Excellent
- 1☐ Very good
- 2☐ Good
- 3☐ Fair
- 4☐ Poor

26. **What is your ethnicity?** *(Please mark one box)*

- 1☐ Hispanic / Latino
- 0☐ Non- Hispanic / Non- Latino

27. **What is your race?** *(Please mark ALL that apply)*

- 1☐ Asian
- 1☐ Black / African-American
- 1☐ Native American / Alaska Native
- 1☐ Native Hawaiian / Samoan / Pacific Islander
- 1☐ White
- 1☐ Other: *please specify* ________________________________

Thank you for taking time to complete this survey.
If you have any comments, please feel free to add them to the space below, or call (206) 744-XXXX to talk with our study team. Thank you again for your help.

© 2022 Lee RY et al. *JAMA Network Open.*

(b) Patient baseline questionnaire for intervention arm

P project to
I improve
C communication about
S serious
I illness
Pilot Study P

Project to Improve Communication about Serious Illness

Thank you for participating in our study to improve communication among doctors, their patients and patients' families.

By improving communication, we hope that we will help patients receive the care they want. Please feel free to skip any questions that you do not want to answer or that you feel do not apply to you. All of your answers are confidential. We will use some of these answers to create the Jumpstart forms for you and your doctor.

If you have questions or need assistance to complete this questionnaire, a member of our research staff would be glad to help you. As you complete the survey, please feel free to add any comments to the margins of the pages or to the space at the end of the survey, or call (206) 744-XXXX to talk with a member of our study team.

Thank you very much. We appreciate your participation.

Please fill in today's date: _________ / _________ /_________
Month Day Year

TALKING ABOUT HEALTH CARE

We are interested in knowing about discussions you may have had with your doctors concerning the medical care you would want if you were very sick. These discussions might include whether you would want to receive care such as ICU care, CPR or breathing machines, a nursing home, or hospice care.

1. <u>In the past, before this hospitalization</u>, have you ever discussed with any of your doctors, in a face-to-face discussion, the kind of medical care you would want if you were too sick to speak for yourself? *(Please mark one box)*

- 1 ☐ Yes
- 0 ☐ No
- 9 ☐ I don't know

2. <u>During this current hospital stay</u>, have you discussed with any of the hospital doctors, in a face-to-face discussion, the kind of medical care you would want if you were too sick to speak for yourself? *(Please mark one box)*

- 1 ☐ Yes
- 0 ☐ No
- 9 ☐ I don't know

3. Would you like to have a discussion/additional discussions of this type with any of the hospital doctors? *(Please mark one box)*

- 1 ☐ Yes
- 0 ☐ No
- 9 ☐ I don't know

Please continue on to the next page

© 2022 Lee RY et al. *JAMA Network Open.*

CHOOSING CARE

We are also interested in the kind of care you prefer and the kind of care you are now receiving.

4. If you had to make a choice at this time, would you prefer a plan of medical care that focuses on <u>extending your life as much as possible</u>, even if it means having more pain and discomfort, or would you want a plan of medical care that focuses on <u>relieving your pain and discomfort as much as possible</u>, even if that means not living as long? *(Please mark one box)*

0 ☐ Extending life, even if it means having more pain and discomfort

1 ☐ Relieving pain and discomfort as much as possible, even if that means not living as long

9 ☐ I'm not sure which I would choose

5. Using those same categories, which of the following best describes the focus of the medical care you are currently receiving? *(Please mark one box)*

0 ☐ Extending life, even if it means having more pain and discomfort

1 ☐ Relieving pain and discomfort as much as possible, even if that means not living as long

9 ☐ I don't know, not sure

Please continue on to the next page

For these next questions, we would like to ask you about your preferences about CPR, a procedure that is administered if a person's heart stops. CPR, or cardiopulmonary resuscitation, consists of electric shocks to the heart, pumping on the chest, and help with breathing. Possible side effects of CPR include broken ribs and memory loss. It is important to realize that, for most people, CPR doesn't work and they do not survive the attempt of CPR.

6. In your <u>current health</u>, would you want CPR if your heart were to stop beating? *(Please mark one box)*

0 ☐ Definitely No

1 ☐ Probably No

2 ☐ Probably Yes

3 ☐ Definitely Yes

7. If you were permanently confined to bed and <u>dependent on others</u> for all your care, would you want CPR if your heart were to stop? *(Please mark one box)*

0 ☐ Definitely No

1 ☐ Probably No

2 ☐ Probably Yes

3 ☐ Definitely Yes

Please continue on to the next page

© 2022 Lee RY et al. *JAMA Network Open.*

TALKING ABOUT CARE: WHAT MAKES TALKING HARDER?

Which, if any, of the following reasons might make it hard for you to talk with any of your doctors about care that you would choose if you were too sick to speak for yourself? Please mark each answer that applies to you.

Barriers	Yes, this applies to me (True)
8a. I don't know what kind of care I would want if I were too sick to speak for myself.	1☐
8b. I'm not ready to talk about the care I would want if I were too sick to speak for myself.	1☐
8c. I don't like to talk about getting very sick.	1☐
8d. My doctor never seems to have the time to talk about issues like end-of-life care.	1☐
8e. I would rather concentrate on staying alive than talk about death.	1☐
8f. I feel that talking about death can bring death closer.	1☐
8g. I have a living will, and that means I don't need to talk with my doctor about the care I would want if I were too sick to speak for myself.	1☐
8h. My ideas about the kind of medical care I want change at different times.	1☐
8i. I have <u>not</u> felt sick enough to talk with my doctor about end-of-life care.	1☐
8j. I'm not sure which doctor would be taking care of me if I were too sick to speak for myself.	1☐
9. If any of these reasons apply to you, which ONE is the <u>main barrier</u> that makes it hard to talk with your doctor about care you might choose if you were too sick to speak for yourself? *Please fill in the number of that item in this box.* →	

Please continue on to the next page

TALKING ABOUT CARE: WHAT MAKES TALKING EASIER?

People also have a number of reasons for wanting to talk about care that they might choose if they were to become too sick to speak for themselves. Which, if any, of the following reasons might make you <u>want</u> to discuss care choices with any of your doctors? Please mark each answer that applies to you.

Facilitators	Yes, this applies to me (True)
10a. I <u>have</u> been very sick in the past so it is easier to talk about.	1☐
10b. I have had family or friends who have died so it is easier to talk about.	1☐
10c. I worry about the quality of my life in the future.	1☐
10d. I worry that I could be a burden on my friends and family if I were to become too sick to speak for myself.	1☐
11. If any of these reasons apply to you, which ONE is the <u>main reasons</u> that makes it easier to talk with your doctor about care you might choose? *Please fill in the number of that item in this box.* →	

Please continue on to the next page

© 2022 Lee RY et al. *JAMA Network Open.*

YOUR FEELINGS

Please mark the box of the answer that best describes how <u>you</u> have been feeling during the LAST WEEK. Don't think too long over your replies; your immediate answer is best.

12. I feel tense or 'wound up':

- 3 ☐ Most of the time
- 2 ☐ A lot of the time
- 1 ☐ From time to time, occasionally
- 0 ☐ Not at all

13. I still enjoy the things I used to enjoy:

- 0 ☐ Definitely as much
- 1 ☐ Not quite so much
- 2 ☐ Only a little
- 3 ☐ Hardly at all

14. I get a sort of frightened feeling as if something awful is about to happen:

- 3 ☐ Very definitely and quite badly
- 2 ☐ Yes, but not too badly
- 1 ☐ A little, but it doesn't worry me
- 0 ☐ Not at all

15. I can laugh and see the funny side of things:

- 0 ☐ As much as I always could
- 1 ☐ Not quite so much now
- 2 ☐ Definitely not so much now
- 3 ☐ Not at all

16. Worrying thoughts go through my mind:

- 3 ☐ A great deal of the time
- 2 ☐ A lot of the time
- 1 ☐ From time to time, but not too often
- 0 ☐ Only occasionally

17. I feel cheerful:

- 3 ☐ Not at all
- 2 ☐ Not often
- 1 ☐ Sometimes
- 0 ☐ Most of the time

18. I can sit at ease and feel relaxed:

- 0 ☐ Definitely
- 1 ☐ Usually
- 2 ☐ Not often
- 3 ☐ Not at all

19. I feel as if I am slowed down:

- 3 ☐ Nearly all the time
- 2 ☐ Very often
- 1 ☐ Sometimes
- 0 ☐ Not at all

© 2022 Lee RY et al. *JAMA Network Open.*

20. **I get a sort of frightened feeling like 'butterflies' in the stomach:**

- 0 ☐ Not at all
- 1 ☐ Occasionally
- 2 ☐ Quite Often
- 3 ☐ Very Often

21. **I have lost interest in my appearance:**

- 3 ☐ Definitely
- 2 ☐ I don't take as much care as I should
- 1 ☐ I may not take quite as much care
- 0 ☐ I take just as much care as ever

22. **I feel restless as if I have to be on the move:**

- 3 ☐ Very much indeed
- 2 ☐ Quite a lot
- 1 ☐ Not very much
- 0 ☐ Not at all

23. **I look forward with enjoyment to things:**

- 0 ☐ As much as I ever did
- 1 ☐ Rather less than I used to
- 2 ☐ Definitely less than I used to
- 3 ☐ Hardly at all

24. **I get sudden feelings of panic:**

- 3 ☐ Very often indeed
- 2 ☐ Quite often
- 1 ☐ Not very often
- 0 ☐ Not at all

25. **I can enjoy a good book or radio or TV program:**

- 0 ☐ Often
- 1 ☐ Sometimes
- 2 ☐ Not often
- 3 ☐ Very seldom

26. **In the past week, how often have you felt upset or irritable?** *(Please mark one box)*

- 1 ☐ Never
- 2 ☐ Rarely
- 3 ☐ Sometimes
- 4 ☐ Often
- 5 ☐ Always

27. **In the last week, how often have you felt depressed?** *(Please mark one box)*

- 1 ☐ Never
- 2 ☐ Rarely
- 3 ☐ Sometimes
- 4 ☐ Often
- 5 ☐ Constantly

© 2022 Lee RY et al. *JAMA Network Open.*

28. In the last week, how often have you felt anxious? *(Please mark one box)*

1☐ Never
2☐ Rarely
3☐ Sometimes
4☐ Often
5☐ Constantly

ABOUT YOU

Lastly, we would like to ask a few questions about you. These questions help us describe the people who are participating in the study.

29. What is your current marital status? *(Please mark one box)*

0☐ Single or never married
1☐ Married or living with partner
2☐ Divorced or separated
3☐ Widowed

30. What is the highest level of schooling that you have completed? *(Please mark one box)*

0☐ 8th grade or less
1☐ Some high school
2☐ High school diploma or GED
3☐ Some college or trade school
4☐ 4-year college degree (ex. BA or BS)
5☐ Some graduate school
6☐ Graduate degree (ex. MA, MS, PhD, MD)

31. In general, would you say your health is: *(Please mark one box)*

0☐ Excellent
1☐ Very good
2☐ Good
3☐ Fair
4☐ Poor

32. What is your ethnicity? *(Please mark one box)*

1☐ Hispanic / Latino
0☐ Non- Hispanic / Non- Latino

33. What is your race? *(Please mark ALL that apply)*

1☐ Asian
1☐ Black / African-American
1☐ Native American / Alaska Native
1☐ Native Hawaiian / Samoan / Pacific Islander
1☐ White
1☐ Other: *please specify* ________________________________

Thank you for taking time to complete this survey.
If you have any comments, please feel free to add them to the space below, or call (206) 744-XXXX to talk with our study team. Thank you again for your help.

© 2022 Lee RY et al. *JAMA Network Open.*

(c) Patient follow-up questionnaire for control arm

P project to
I improve
C communication about
S serious
I illness
Pilot Study P

Project to Improve Communication about Serious Illness

Thank you for participating in our study to improve communication among doctors, their patients and patients' families.

The questions in this booklet include items about discussions with your doctors and your feelings since being in the hospital. Please feel free to skip any questions that you feel do not apply to you or that you do not want to answer. All of your answers are confidential.

If you have questions or need assistance to complete this questionnaire, a member of our study team would be glad to help you. As you complete the survey, please feel free to add any comments to the margins of the pages or to the space at the end of the survey, or call (206) 744-XXXX to talk with a member of our study team.

Thank you very much. We appreciate your participation.

Please fill in today's date: __________ / __________ /__________
Month Day Year

[CG] Usual Care Group – Follow-Up Questionnaire for Patient v.9

TALKING ABOUT HEALTHCARE

This first group of questions is about discussions you may have recently had with the doctor(s) who have been caring for you while you have been in the hospital.

1a. **During this hospital stay, have you discussed with a doctor, in a face-to-face discussion, the kind of medical care you would want if you were too sick to speak for yourself?** *(Please mark one box)*

1 ☐ Yes
0 ☐ No → (GO TO PAGE 3, QUESTION # 2)
9 ☐ I don't know → (GO TO PAGE 3, QUESTION # 2)

1b. If "YES": **To what extent did the discussion(s) meet your needs for information about your medical care?** *(Please mark one box)*

Not at all										Completely
0	1	2	3	4	5	6	7	8	9	10
☐	☐	☐	☐	☐	☐	☐	☐	☐	☐	☐

Please continue on to the next page

© 2022 Lee RY et al. *JAMA Network Open.*

CHOOSING CARE

We are also interested in the kind of care you prefer and the kind of care you are now receiving.

2. If you had to make a choice at this time, would you prefer a plan of medical care that focuses on extending your life as much as possible, even if it means having more pain and discomfort, or would you want a plan of medical care that focuses on relieving your pain and discomfort as much as possible, even if that means not living as long? *(Please mark one box)*

0 ☐ Extending life, even if it means having more pain and discomfort

1 ☐ Relieving pain and discomfort as much as possible, even if that means not living as long

9 ☐ I'm not sure which I would choose

3. Using those same categories, which of the following best describes the focus of the medical care you are currently receiving? *(Please mark one box)*

0 ☐ Extending life, even if it means having more pain and discomfort

1 ☐ Relieving pain and discomfort as much as possible, even if that means not living as long

9 ☐ I don't know, not sure

HOW WELL DID YOUR DOCTOR TALK WITH YOU?

The following questions are about how well the doctor(s) talked with you about your care during this hospital stay. We know that many people think very highly of the doctors. To help us improve communication between doctors and patients, please be critical.

Please rate your hospital doctor(s) on each of the following questions using a scale from 0, "The very worst I could imagine" to 10, "The very best I could imagine." If you cannot rate the doctor on a question because he or she has not done it, please mark the box, "The doctor has not done this." You may also mark the box, "I do not know."

How good was the hospital doctor(s) at: *(Please mark one box for each item)*

4. Talking with you about your feelings concerning the possibility that you might get sicker? *(Please mark one box)*

The very worst I could imagine										The very best I could imagine	*The doctor has not done this*	*I do not know*
0	1	2	3	4	5	6	7	8	9	10	888	999
☐	☐	☐	☐	☐	☐	☐	☐	☐	☐	☐	☐	☐

5. Talking with you about the details concerning the possibility that you might get sicker? *(Please mark one box)*

The very worst I could imagine										The very best I could imagine	*The doctor has not done this*	*I do not know*
0	1	2	3	4	5	6	7	8	9	10	888	999
☐	☐	☐	☐	☐	☐	☐	☐	☐	☐	☐	☐	☐

© 2022 Lee RY et al. *JAMA Network Open.*

How good was the hospital doctor(s)at: *(Please mark one box for each item)*

6. Talking to you about how long you might have to live? *(Please mark one box)*

The very worst I could imagine										The very best I could imagine	*The doctor has not done this*	*I do not know*
0	1	2	3	4	5	6	7	8	9	10	888	999
☐	☐	☐	☐	☐	☐	☐	☐	☐	☐	☐	☐	☐

7. Talking with you about what dying might be like? *(Please mark one box)*

The very worst I could imagine										The very best I could imagine	*The doctor has not done this*	*I do not know*
0	1	2	3	4	5	6	7	8	9	10	888	999
☐	☐	☐	☐	☐	☐	☐	☐	☐	☐	☐	☐	☐

8. Involving you in the decisions about the treatments that you want if you get too sick to speak for yourself? *(Please mark one box)*

The very worst I could imagine										The very best I could imagine	*The doctor has not done this*	*I do not know*
0	1	2	3	4	5	6	7	8	9	10	888	999
☐	☐	☐	☐	☐	☐	☐	☐	☐	☐	☐	☐	☐

9. Asking about the things in life that are important to you? *(Please mark one box)*

The very worst I could imagine										The very best I could imagine	*The doctor has not done this*	*I do not know*
0	1	2	3	4	5	6	7	8	9	10	888	999
☐	☐	☐	☐	☐	☐	☐	☐	☐	☐	☐	☐	☐

How good was the hospital doctor(s) at: *(Please mark one box for each item)*

10. Asking about your spiritual or religious beliefs? *(Please mark one box)*

The very worst I could imagine										The very best I could imagine	*The doctor has not done this*	*I do not know*
0	1	2	3	4	5	6	7	8	9	10	888	999
☐	☐	☐	☐	☐	☐	☐	☐	☐	☐	☐	☐	☐

11. How much do you agree or disagree with the following statement? *(Please mark one box)*
"The discussions I had with my hospital doctor(s) during the current hospital stay covered all of the important topics that needed to be discussed."

- 1 ☐ Strongly disagree
- 2 ☐ Disagree
- 3 ☐ Neither agree nor disagree
- 4 ☐ Agree
- 5 ☐ Strongly agree
- 8 ☐ N/a: no discussion with the doctor(s) occurred

12. How satisfied are you with the quality of the conversations your hospital doctor(s) had with you during the current hospital stay? *(Please mark one box)*

- 1 ☐ Extremely dissatisfied
- 2 ☐ Dissatisfied
- 3 ☐ Neither satisfied nor dissatisfied
- 4 ☐ Satisfied
- 5 ☐ Extremely satisfied
- 8 ☐ N/a: no conversation with the doctor(s) occurred

© 2022 Lee RY et al. *JAMA Network Open.*

13. How would you rate the overall helpfulness of your discussions with your hospital doctor(s) during the current hospital stay? *(Please mark one box)*

- 1 ☐ Poor (several needed topics were not covered or were covered poorly)
- 2 ☐ Fair
- 3 ☐ Good
- 4 ☐ Very good
- 5 ☐ Outstanding (couldn't have been better)
- 8 ☐ N/a: no discussion with the doctor(s) occurred

14. How would you rate the discussions you had with your hospital doctor(s) during the current hospital stay? Please check the number that corresponds with your rating, or check the box that indicates that no discussions occurred. *(Please mark one box)*

The very worst I could imagine										The very best I could imagine	*No discussions occurred*
0	1	2	3	4	5	6	7	8	9	10	888
☐	☐	☐	☐	☐	☐	☐	☐	☐	☐	☐	☐

Please continue on to the next page

EXPERIENCES DURING YOUR HOSPITALIZATION

Please answer these questions about your experiences during this hospitalization.

15. How much of the time has it been difficult to make important medical decisions? *(Please mark one box)*

None										A lot
0	1	2	3	4	5	6	7	8	9	10
☐	☐	☐	☐	☐	☐	☐	☐	☐	☐	☐

16. How much of the time do you suffer from physical symptoms (e.g. pain, shortness of breath, etc.)? *(Please mark one box)*

None										A lot
0	1	2	3	4	5	6	7	8	9	10
☐	☐	☐	☐	☐	☐	☐	☐	☐	☐	☐

17. How much of the time do you feel stressed out or depressed? *(Please mark one box)*

None										A lot
0	1	2	3	4	5	6	7	8	9	10
☐	☐	☐	☐	☐	☐	☐	☐	☐	☐	☐

18. How much of the time is there someone to give you support? *(Please mark one box)*

None										A lot
0	1	2	3	4	5	6	7	8	9	10
☐	☐	☐	☐	☐	☐	☐	☐	☐	☐	☐

19. How much spiritual support have you received in the hospital? *(Please mark one box)*

None										A lot
0	1	2	3	4	5	6	7	8	9	10
☐	☐	☐	☐	☐	☐	☐	☐	☐	☐	☐

© 2022 Lee RY et al. *JAMA Network Open.*

20. **How often do you have regular discussions with the medical team (to talk about the condition, treatments, or your concerns)?** *(Please mark one box)*

None										A lot
0	1	2	3	4	5	6	7	8	9	10
☐	☐	☐	☐	☐	☐	☐	☐	☐	☐	☐

21. **Has information been provided in your preferred language and in a way that respects your cultural beliefs?** *(Please mark one box)*

Not at all										Completely
0	1	2	3	4	5	6	7	8	9	10
☐	☐	☐	☐	☐	☐	☐	☐	☐	☐	☐

22. **How much do you trust the medical information you have received?** *(Please mark one box)*

Not at all										Completely
0	1	2	3	4	5	6	7	8	9	10
☐	☐	☐	☐	☐	☐	☐	☐	☐	☐	☐

23. **Do you have enough information about what to expect with your illness?** *(Please mark one box)*

Not at all										Completely
0	1	2	3	4	5	6	7	8	9	10
☐	☐	☐	☐	☐	☐	☐	☐	☐	☐	☐

24. **How well does your current medical treatment fit with your values?** *(Please mark one box)*

Not at all										Completely
0	1	2	3	4	5	6	7	8	9	10
☐	☐	☐	☐	☐	☐	☐	☐	☐	☐	☐

25. **How sure are you about your beliefs about life support?** *(Please mark one box)*

Not at all										Completely
0	1	2	3	4	5	6	7	8	9	10
☐	☐	☐	☐	☐	☐	☐	☐	☐	☐	☐

26. **How much of a financial hardship is your illness for you or your family?** *(Please mark one box)*

None										A lot
0	1	2	3	4	5	6	7	8	9	10
☐	☐	☐	☐	☐	☐	☐	☐	☐	☐	☐

YOUR FEELINGS

Please mark the box of the answer that best describes how you have been feeling <u>during the LAST WEEK</u>. Don't think too long over your replies; your immediate answer is best.

27. **I feel tense or 'wound up':**

- 3 ☐ Most of the time
- 2 ☐ A lot of the time
- 1 ☐ From time to time, occasionally
- 0 ☐ Not at all

28. **I still enjoy the things I used to enjoy:**

- 0 ☐ Definitely as much
- 1 ☐ Not quite so much
- 2 ☐ Only a little
- 3 ☐ Hardly at all

© 2022 Lee RY et al. *JAMA Network Open.*

29. I get a sort of frightened feeling as if something awful is about to happen:

- 3 ☐ Very definitely and quite badly
- 2 ☐ Yes, but not too badly
- 1 ☐ A little, but it doesn't worry me
- 0 ☐ Not at all

30. I can laugh and see the funny side of things:

- 0 ☐ As much as I always could
- 1 ☐ Not quite so much now
- 2 ☐ Definitely not so much now
- 3 ☐ Not at all

31. Worrying thoughts go through my mind:

- 3 ☐ A great deal of the time
- 2 ☐ A lot of the time
- 1 ☐ From time to time, but not too often
- 0 ☐ Only occasionally

32. I feel cheerful:

- 3 ☐ Not at all
- 2 ☐ Not often
- 1 ☐ Sometimes
- 0 ☐ Most of the time

33. I can sit at ease and feel relaxed:

- 0 ☐ Definitely
- 1 ☐ Usually
- 2 ☐ Not often
- 3 ☐ Not at all

34. I feel as if I am slowed down:

- 3 ☐ Nearly all the time
- 2 ☐ Very often
- 1 ☐ Sometimes
- 0 ☐ Not at all

35. I get a sort of frightened feeling like 'butterflies' in the stomach:

- 0 ☐ Not at all
- 1 ☐ Occasionally
- 2 ☐ Quite Often
- 3 ☐ Very Often

36. I have lost interest in my appearance:

- 3 ☐ Definitely
- 2 ☐ I don't take as much care as I should
- 1 ☐ I may not take quite as much care
- 0 ☐ I take just as much care as ever

© 2022 Lee RY et al. *JAMA Network Open.*

37. **I feel restless as if I have to be on the move:**

- 3☐ Very much indeed
- 2☐ Quite a lot
- 1☐ Not very much
- 0☐ Not at all

38. **I look forward with enjoyment to things:**

- 0☐ As much as I ever did
- 1☐ Rather less than I used to
- 2☐ Definitely less than I used to
- 3☐ Hardly at all

39. **I get sudden feelings of panic:**

- 3☐ Very often indeed
- 2☐ Quite often
- 1☐ Not very often
- 0☐ Not at all

40. **I can enjoy a good book or radio or TV program:**

- 0☐ Often
- 1☐ Sometimes
- 2☐ Not often
- 3☐ Very seldom

41. **In the past week, how often have you felt upset or irritable?** *(Please mark one box)*

- 1☐ Never
- 2☐ Rarely
- 3☐ Sometimes
- 4☐ Often
- 5☐ Always

42. **In the last week, how often have you felt depressed?** *(Please mark one box)*

- 1☐ Never
- 2☐ Rarely
- 3☐ Sometimes
- 4☐ Often
- 5☐ Constantly

43. **In the last week, how often have you felt anxious?** *(Please mark one box)*

- 1☐ Never
- 2☐ Rarely
- 3☐ Sometimes
- 4☐ Often
- 5☐ Constantly

44. **Lastly, how much of a burden on you was completing the study questionnaires, on a scale where 0 = "No burden at all" and 10 = "Great burden"?** *(Please mark one box)*

No burden at all					Moderate burden					Great burden
0	1	2	3	4	5	6	7	8	9	10
☐	☐	☐	☐	☐	☐	☐	☐	☐	☐	☐

© 2022 Lee RY et al. *JAMA Network Open.*

Thank you for taking the time to complete this survey.
If you have any comments, please feel free to add them to the space below, or call (206) 744-XXXX to talk with our study team. Thank you again for your help.

© 2022 Lee RY et al. *JAMA Network Open.*

(d) Patient follow-up questionnaire for intervention arm

P project to
I improve
C communication about
S serious
I illness
Pilot Study **P**

Project to Improve Communication about Serious Illness

Thank you for participating in our study to improve communication among doctors, their patients and patients' families.

The questions in this booklet include items about discussions with your doctors and your feelings since being in the hospital. Please feel free to skip any questions that you feel do not apply to you or that you do not want to answer. All of your answers are confidential.

If you have questions or need assistance to complete this questionnaire, a member of our study team would be glad to help you. As you complete the survey, please feel free to add any comments to the margins of the pages or to the space at the end of the survey, or call (206) 744-XXXX to talk with a member of our study team.

Thank you very much. We appreciate your participation.

Please fill in today's date: __________ / __________ /__________
Month Day Year

[IG] Intervention Group – Follow-Up Questionnaire for Patient v.9

TALKING ABOUT HEALTHCARE

This first group of questions is about discussions you may have recently had with any of the doctors who have been caring for you <u>while you have been in the hospital</u>.

1a. **<u>During this hospital stay</u>, have you discussed with any of the doctors, in a face-to-face discussion, the kind of medical care you would want if you were too sick to speak for yourself?** *(Please mark one box)*

- 1 ☐ Yes
- 0 ☐ No → (GO TO PAGE 3, QUESTION # 2)
- 9 ☐ I don't know → (GO TO PAGE 3, QUESTION #2)

1b. If "YES": **To what extent did the discussion(s) meet your needs for information about your medical care?** *(Please mark one box)*

Not at all										Completely
0	1	2	3	4	5	6	7	8	9	10
☐	☐	☐	☐	☐	☐	☐	☐	☐	☐	☐

Please continue on to the next page

© 2022 Lee RY et al. *JAMA Network Open.*

CHOOSING CARE

We are also interested in the kind of care you prefer and the kind of care you are now receiving.

2. If you had to make a choice at this time, would you prefer a plan of medical care that focuses on extending your life as much as possible, even if it means having more pain and discomfort, or would you want a plan of medical care that focuses on relieving your pain and discomfort as much as possible, even if that means not living as long? *(Please mark one box)*

- 0 ☐ Extending life, even if it means having more pain and discomfort
- 1 ☐ Relieving pain and discomfort as much as possible, even if that means not living as long
- 9 ☐ I'm not sure which I would choose

3. Using those same categories, which of the following best describes the focus of the medical care you are currently receiving? *(Please mark one box)*

- 0 ☐ Extending life, even if it means having more pain and discomfort
- 1 ☐ Relieving pain and discomfort as much as possible, even if that means not living as long
- 9 ☐ I don't know, not sure

Please continue on to the next page

HOW WELL DID YOUR DOCTOR TALK WITH YOU?

The following questions are about how well the doctor(s) talked with you about your care during this hospital stay. We know that many people think very highly of their doctors. To help us improve communication between doctors and patients, please be critical.

Please rate your hospital doctor(s) on each of the following questions using a scale from 0, "The very worst I could imagine" to 10, "The very best I could imagine." If you cannot rate the doctor on a question because he or she has not done it, please mark the box, "The doctor has not done this." You may also mark the box, "I do not know."

How good was your hospital doctor at: *(Please mark one box for each item)*

4. Talking with you about your feelings concerning the possibility that you might get sicker? *(Please mark one box)*

The very worst I could imagine										The very best I could imagine	*The doctor has not done this*	*I do not know*
0	1	2	3	4	5	6	7	8	9	10	888	999
☐	☐	☐	☐	☐	☐	☐	☐	☐	☐	☐	☐	☐

5. Talking with you about the details concerning the possibility that you might get sicker? *(Please mark one box)*

The very worst I could imagine										The very best I could imagine	*The doctor has not done this*	*I do not know*
0	1	2	3	4	5	6	7	8	9	10	888	999
☐	☐	☐	☐	☐	☐	☐	☐	☐	☐	☐	☐	☐

© 2022 Lee RY et al. *JAMA Network Open.*

How good was your hospital doctor at: *(Please mark one box for each item)*

6. Talking to you about how long you might have to live? *(Please mark one box)*

The very worst I could imagine										The very best I could imagine	*The doctor has not done this*	*I do not know*
0	1	2	3	4	5	6	7	8	9	10	888	999
☐	☐	☐	☐	☐	☐	☐	☐	☐	☐	☐	☐	☐

7. Talking with you about what dying might be like? *(Please mark one box)*

The very worst I could imagine										The very best I could imagine	*The doctor has not done this*	*I do not know*
0	1	2	3	4	5	6	7	8	9	10	888	999
☐	☐	☐	☐	☐	☐	☐	☐	☐	☐	☐	☐	☐

8. Involving you in the decisions about the treatments that you want if you get too sick to speak for yourself? *(Please mark one box)*

The very worst I could imagine										The very best I could imagine	*The doctor has not done this*	*I do not know*
0	1	2	3	4	5	6	7	8	9	10	888	999
☐	☐	☐	☐	☐	☐	☐	☐	☐	☐	☐	☐	☐

9. Asking about the things in life that are important to you? ***(Please mark one box)***

The very worst I could imagine										The very best I could imagine	*The doctor has not done this*	*I do not know*
0	1	2	3	4	5	6	7	8	9	10	888	999
☐	☐	☐	☐	☐	☐	☐	☐	☐	☐	☐	☐	☐

How good was your hospital doctor at: *(Please mark one box for each item)*

10. Asking about your spiritual or religious beliefs? *(Please mark one box)*

The very worst I could imagine										The very best I could imagine	*The doctor has not done this*	*I do not know*
0	1	2	3	4	5	6	7	8	9	10	888	999
☐	☐	☐	☐	☐	☐	☐	☐	☐	☐	☐	☐	☐

11. How much do you agree or disagree with the following statement? *(Please mark one box)*

"The discussions I had with my hospital doctor(s) during the current hospital stay covered all of the important topics that needed to be discussed."

1 ☐ Strongly disagree
2 ☐ Disagree
3 ☐ Neither agree nor disagree
4 ☐ Agree
5 ☐ Strongly agree
8 ☐ N/a: no discussion with the doctor(s) occurred

12. How satisfied are you with the quality of the conversations your hospital doctor(s) had with you during the current hospital stay? *(Please mark one box)*

1 ☐ Extremely dissatisfied
2 ☐ Dissatisfied
3 ☐ Neither satisfied nor dissatisfied
4 ☐ Satisfied
5 ☐ Extremely satisfied
8 ☐ N/a: no conversation with the doctor(s) occurred

© 2022 Lee RY et al. *JAMA Network Open.*

13. How would you rate the overall helpfulness of your discussions with your hospital doctor(s) during the current hospital stay? *(Please mark one box)*

- 1☐ Poor (several needed topics were not covered or were covered poorly)
- 2☐ Fair
- 3☐ Good
- 4☐ Very good
- 5☐ Outstanding (couldn't have been better)
- 8☐ N/a: no discussion with the doctor(s) occurred

14. How would you rate the discussions you had with your hospital doctor(s) during the current hospital stay? Please check the number that corresponds with your rating, or check the box that indicates that no discussions occurred. *(Please mark one box)*

The very worst I could imagine										The very best I could imagine	*No discussions occurred*
0	1	2	3	4	5	6	7	8	9	10	888
☐	☐	☐	☐	☐	☐	☐	☐	☐	☐	☐	☐

THE JUMPSTART FORM: DID YOU LIKE IT?

Please help us improve the way patients and families use the Jumpstart form to talk with their doctors. We are interested in your honest opinions, whether positive or negative. We also welcome your comments and suggestions.

15. Did you use the Jumpstart form? *(Please mark one box)*

- 1☐ Yes
- 0☐ No → GO TO PAGE 9, QUESTION #20
- 9☐ I'm not sure; I don't know what the Jumpstart form is → GO TO PAGE 9, QUESTION #20

16. Did the Jumpstart form help you <u>start a conversation</u> with your hospital doctor(s)? *(Please mark one box)*

- 0☐ Definitely No
- 1☐ Probably No
- 2☐ Probably Yes
- 3☐ Definitely Yes

17. Did the Jumpstart form help you <u>get the information</u> you wanted from your doctor? *(Please mark one box)*

- 0☐ Definitely No
- 1☐ Probably No
- 2☐ Probably Yes
- 3☐ Definitely Yes

18. Did the Jumpstart form help you <u>make decisions</u> for your healthcare? *(Please mark one box)*

- 0☐ Definitely No
- 1☐ Probably No
- 2☐ Probably Yes
- 3☐ Definitely Yes

19. Would you recommend the Jumpstart form to other patients? *(Please mark one box)*

- 0☐ Definitely No
- 1☐ Probably No
- 2☐ Probably Yes
- 3☐ Definitely Yes

© 2022 Lee RY et al. *JAMA Network Open.*

EXPERIENCES DURING YOUR HOSPITALIZATION

Please answer these questions about your experiences during this hospitalization.

20. How much of the time has it been difficult to make important medical decisions? *(Please mark one box)*

None										A lot
0	1	2	3	4	5	6	7	8	9	10
☐	☐	☐	☐	☐	☐	☐	☐	☐	☐	☐

21. How much of the time do you suffer from physical symptoms (e.g. pain, shortness of breath, etc.)? *(Please mark one box)*

None										A lot
0	1	2	3	4	5	6	7	8	9	10
☐	☐	☐	☐	☐	☐	☐	☐	☐	☐	☐

22. How much of the time do you feel stressed out or depressed? *(Please mark one box)*

None										A lot
0	1	2	3	4	5	6	7	8	9	10
☐	☐	☐	☐	☐	☐	☐	☐	☐	☐	☐

23. How much of the time is there someone to give you support? *(Please mark one box)*

None										A lot
0	1	2	3	4	5	6	7	8	9	10
☐	☐	☐	☐	☐	☐	☐	☐	☐	☐	☐

24. How much spiritual support have you received in the hospital? *(Please mark one box)*

None										A lot
0	1	2	3	4	5	6	7	8	9	10
☐	☐	☐	☐	☐	☐	☐	☐	☐	☐	☐

25. How often do you have regular discussions with the medical team (to talk about the condition, treatments, or your concerns)? *(Please mark one box)*

None										A lot
0	1	2	3	4	5	6	7	8	9	10
☐	☐	☐	☐	☐	☐	☐	☐	☐	☐	☐

26. Has information been provided in your preferred language and in a way that respects your cultural beliefs? *(Please mark one box)*

Not at all										Completely
0	1	2	3	4	5	6	7	8	9	10
☐	☐	☐	☐	☐	☐	☐	☐	☐	☐	☐

27. How much do you trust the medical information you have received? *(Please mark one box)*

Not at all										Completely
0	1	2	3	4	5	6	7	8	9	10
☐	☐	☐	☐	☐	☐	☐	☐	☐	☐	☐

28. Do you have enough information about what to expect with your illness? *(Please mark one box)*

Not at all										Completely
0	1	2	3	4	5	6	7	8	9	10
☐	☐	☐	☐	☐	☐	☐	☐	☐	☐	☐

29. How well does your current medical treatment fit with your values? *(Please mark one box)*

Not at all										Completely
0	1	2	3	4	5	6	7	8	9	10
☐	☐	☐	☐	☐	☐	☐	☐	☐	☐	☐

30. How sure are you about your beliefs about life support? *(Please mark one box)*

Not at all										Completely
0	1	2	3	4	5	6	7	8	9	10
☐	☐	☐	☐	☐	☐	☐	☐	☐	☐	☐

© 2022 Lee RY et al. *JAMA Network Open.*

31. How much of a financial hardship is your illness for you or your family? *(Please mark one box)*

None										A lot
0	1	2	3	4	5	6	7	8	9	10
☐	☐	☐	☐	☐	☐	☐	☐	☐	☐	☐

YOUR FEELINGS

Please mark the box of the answer that best describes how you have been feeling during the LAST WEEK. Don't think too long over your replies; your immediate answer is best.

32. I feel tense or 'wound up':

- 3 ☐ Most of the time
- 2 ☐ A lot of the time
- 1 ☐ From time to time, occasionally
- 0 ☐ Not at all

33. I still enjoy the things I used to enjoy:

- 0 ☐ Definitely as much
- 1 ☐ Not quite so much
- 2 ☐ Only a little
- 3 ☐ Hardly at all

34. I get a sort of frightened feeling as if something awful is about to happen:

- 3 ☐ Very definitely and quite badly
- 2 ☐ Yes, but not too badly
- 1 ☐ A little, but it doesn't worry me
- 0 ☐ Not at all

35. I can laugh and see the funny side of things:

- 0 ☐ As much as I always could
- 1 ☐ Not quite so much now
- 2 ☐ Definitely not so much now
- 3 ☐ Not at all

36. Worrying thoughts go through my mind:

- 3 ☐ A great deal of the time
- 2 ☐ A lot of the time
- 1 ☐ From time to time, but not too often
- 0 ☐ Only occasionally

37. I feel cheerful:

- 3 ☐ Not at all
- 2 ☐ Not often
- 1 ☐ Sometimes
- 0 ☐ Most of the time

38. I can sit at ease and feel relaxed:

- 0 ☐ Definitely
- 1 ☐ Usually
- 2 ☐ Not often
- 3 ☐ Not at all

© 2022 Lee RY et al. *JAMA Network Open.*

39. I feel as if I am slowed down:

- 3 ☐ Nearly all the time
- 2 ☐ Very often
- 1 ☐ Sometimes
- 0 ☐ Not at all

40. I get a sort of frightened feeling like 'butterflies' in the stomach:

- 0 ☐ Not at all
- 1 ☐ Occasionally
- 2 ☐ Quite Often
- 3 ☐ Very Often

41. I have lost interest in my appearance:

- 3 ☐ Definitely
- 2 ☐ I don't take as much care as I should
- 1 ☐ I may not take quite as much care
- 0 ☐ I take just as much care as ever

42. I feel restless as if I have to be on the move:

- 3 ☐ Very much indeed
- 2 ☐ Quite a lot
- 1 ☐ Not very much
- 0 ☐ Not at all

43. I look forward with enjoyment to things:

- 0 ☐ As much as I ever did
- 1 ☐ Rather less than I used to
- 2 ☐ Definitely less than I used to
- 3 ☐ Hardly at all

44. I get sudden feelings of panic:

- 3 ☐ Very often indeed
- 2 ☐ Quite often
- 1 ☐ Not very often
- 0 ☐ Not at all

45. I can enjoy a good book or radio or TV program:

- 0 ☐ Often
- 1 ☐ Sometimes
- 2 ☐ Not often
- 3 ☐ Very seldom

46. In the past week, how often have you felt upset or irritable? *(Please mark one box)*

- 1 ☐ Never
- 2 ☐ Rarely
- 3 ☐ Sometimes
- 4 ☐ Often
- 5 ☐ Always

© 2022 Lee RY et al. *JAMA Network Open.*

47. In the last week, how often have you felt depressed? *(Please mark one box)*

1 ☐ Never
2 ☐ Rarely
3 ☐ Sometimes
4 ☐ Often
5 ☐ Constantly

48. In the last week, how often have you felt anxious? *(Please mark one box)*

1 ☐ Never
2 ☐ Rarely
3 ☐ Sometimes
4 ☐ Often
5 ☐ Constantly

49. Lastly, how much of a burden on you was completing the study questionnaires, on a scale where 0 = "No burden at all" and 10 = "Great burden"? *(Please mark one box)*

No burden at all					Moderate burden					Great burden
0	1	2	3	4	5	6	7	8	9	10
☐	☐	☐	☐	☐	☐	☐	☐	☐	☐	☐

Thank you for taking time to complete this survey.
If you have any comments, please feel free to add them to the space below, or call (206) 744-XXXX to talk with our study team. Thank you again for your help.

© 2022 Lee RY et al. *JAMA Network Open.*

(e) Follow-up questionnaire for clinicians that received the Jumpstart Guide

Page 1

CLINICIAN FOLLOW-UP QUESTIONNAIRE

Thank you for your willingness to participate in this study of the Jumpstart form. In the following questionnaire, we ask how well the Jumpstart form worked for you.

During this patient's hospitalization, did you talk with the patient about his/her goals of care?	○ Yes<br>○ No<br>○ Unsure
If No": What were the reasons that you didn't talk with the patient about his/her goals of care? (Mark all that apply)	☐ Patient was unable to communicate<br>☐ No t me during the hosp tal stay<br>☐ Topics were addressed previously, didn't need to revisit<br>☐ Not appropriate for this patient<br>☐ Other
Other reason not discussed text	______________
If not appropriate" for this patient, please select a reason: (Mark all that apply)	☐ Patient not ready<br>☐ Another clinician is responsible for these discussions<br>☐ I'm not comfortable discussing goals of care with this patient<br>☐ Other
Other reason not appropriate text	______________
During this hospitalization, did you talk with this patient's family or surrogate about the patient's goals of care?	○ Yes<br>○ No<br>○ Unsure
If No": What were the reasons that you didn't talk with this patient's family or surrogate about his/her goals of care? (Mark all that apply)	☐ Patient was able to communicate, so talked to patient instead<br>☐ Family/surrogate was unavailable<br>☐ No t me during the hosp tal stay<br>☐ Topics were addressed previously, didn't need to revisit<br>☐ Not appropriate for this patient<br>☐ Other
Other reason not discussed with family text	______________
If "not appropriate" for this family/surrogate, please select a reason: (Mark all that apply)	☐ Family/Surrogate not ready<br>☐ Another clinician is responsible for these discussions<br>☐ I'm not comfortable discussing goals of care with this family/surrogate<br>☐ Other
Other reason not appropriate (family) text	______________

Page 2

During th s patient's hospitalization, did you receive a "Jumpstart form?	○ Yes<br>○ No<br>○ Unsure
Did the Jumpstart form help you start a goals-of-care discussion with your patient or his/her family member/surrogate?	○ Definitely No<br>○ Probably No<br>○ Probably Yes<br>○ Definitely Yes<br>○ N/A didn't use the Jumpstart form
Would you recommend the Jumpstart form to other clinicians?	○ Definitely No<br>○ Probably No<br>○ Probably Yes<br>○ Definitely Yes
What year were you born?	______________
Please provide us with any feedback you would ike to share about the Jumpstart form:	______________

This questionnaire was electronically administered using REDCap.[114]

© 2022 Lee RY et al. *JAMA Network Open.*

eFigure 1. Sample Patient- and Clinician-Facing Jumpstart Guides.

(a) Patient-facing Jumpstart Guide

Jumpstart Guide

A program to promote goals of care discussions

If you feel unsure or nervous to talk with the hospital doctor about your medical care:
That's ok! The hospital doctor is interested in hearing your thoughts and worries about your medical care. Many people think the doctor might not have time or might know best – but remember you are the expert on what is important in your life. *Show this guide to your hospital doctor to help start a conversation.*

We encourage you to ask the hospital doctor two important questions:

1. Could we talk about what I want if something more serious were to happen *now* in my current health?
2. I am hoping for the best, but I also want to plan if something life-threatening were to happen *in the future*. Could we talk about that?

Here is information from your survey. You can share these answers with your doctor.

**We asked:**	**John Doe, you answered:**
Would you like to talk to any of the hospital doctors about medical care you would want if you should become too sick to speak for yourself?	"Yes."
Do you prefer medical care more focused on extending life or on quality of life?	"I am NOT SURE what I would prefer as the focus of my medical care."
Is your current medical care more focused on extending life or on quality of life?	"I am NOT SURE what the current focus of medical care is that I'm receiving."
Have you thought about whether you would want CPR in your current health?	"If my health is like it is today, I'd probably want CPR."
Have you thought about whether you would want CPR if you were confined to bed and dependent on others?	"If I were permanently confined to bed and dependent on others, I'd definitely not want CPR."

Thanks for using this Jumpstart Guide. We hope this information is helpful.

(b) Clinician-facing Jumpstart Guide

Jumpstart Guide

A program to promote goals of care discussions

What hasn't your patient told you about their goals of care?

The following information is specific to **John Doe, MRN: H1234567**. It's confidential. Give yourself 5-10 minutes—this can be just the start of a discussion. Pick one or two of the topics below—you don't need to address them all!

What the chart contains:

Code Status	Full code	10/15/2019
Advance directive	YES	12/15/2018
DPOA health care	YES	10/20/2018
POLST	NO	

**From your patient's survey:**	**Ideas for what you could say:**
<u>Willingness to talk about goals of care</u><br>"I do not know if I want to talk about my preferences."	"It is helpful for me if I make sure I understand your thoughts about the care you would want if you were to get sicker."
<u>Current goals of care</u><br>"I am NOT SURE whether my current care is focusing on extending life or quality of life."	"Some people prefer care focused on extending life as much as possible; other people prefer care focused on quality of life and comfort. Would it be helpful for me to clarify how I see the focus of your care?"
<u>Barriers to talking about goals</u><br>"I would rather concentrate on staying alive than talk about death."	"Some people find it hard to talk about their healthcare in the future. Would you consider giving it a try for a few minutes? At any time, you can just say, 'Ok, that's enough for today.'"
<u>Preferences for CPR</u><br>"In my current state of health, I PROBABLY DO NOT WANT CPR."	"You indicated you would not want to receive CPR if your heart were to stop beating. Is that correct?" If yes, consider completing a DNAR order now and a POLST form prior to discharge or a Palliative Care consult.

Document a short note

A brief summary and a quote (a few of the patient's words) are enough.
Your colleagues will appreciate it.

© 2022 Lee RY et al. *JAMA Network Open.*

eFigure 2. Matrix of Patient or Surrogate Questionnaire Responses and Clinician Communication Prompts

PICSI-P Matrix for **Clinician-Patient Jumpstart Guide**

Item 1: **Willingness to talk about goals of care**

		**Question 3: Want to discuss / discuss more?**			
		Yes*	No	Don't Know	Skip
**Question 1:** Ever discussed preferences if/when too sick to speak for self?	Yes	"I'm glad you are willing to talk about the care you would want if you were to get sicker. Tell me your thoughts about the Jumpstart Guide."	"It is helpful for me if I make sure I understand your thoughts about the care you would want if you were to get sicker."	"It is helpful for me if I make sure I understand your thoughts about the care you would want if you were to get sicker."	"It is helpful for me if I make sure I understand your thoughts about the care you would want if you were to get sicker."
	No**	"I'm glad you are willing to talk about the care you would want if you were to get sicker. Tell me your thoughts about the Jumpstart Guide."	"It is helpful for me if I make sure I understand your thoughts about the care you would want if you were to get sicker. I know this can be difficult, but it can help make sure you get the care you want."	"It is helpful for me if I make sure I understand your thoughts about the care you would want if you were to get sicker."	"It is helpful for me if I make sure I understand your thoughts about the care you would want if you were to get sicker."
	Don't remember	"I'm glad you are willing to talk about the care you would want if you were to get sicker. Tell me your thoughts about the Jumpstart Guide."	"It is helpful for me if I make sure I understand your thoughts about the care you would want if you were to get sicker. I know this can be difficult, but it can help make sure you get the care you want."	"It is helpful for me if I make sure I understand your thoughts about the care you would want if you were to get sicker."	"It is helpful for me if I make sure I understand your thoughts about the care you would want if you were to get sicker."
	Skip	"I'm glad you are willing to talk about the care you would want if you were to get sicker. Tell me your thoughts about the Jumpstart Guide."	"It is helpful for me if I make sure I understand your thoughts about the care you would want if you were to get sicker. I know this can be difficult, but it can help make sure you get the care you want."	"It is helpful for me if I make sure I understand your thoughts about the care you would want if you were to get sicker."	"It is helpful for me if I make sure I understand your thoughts about the care you would want if you were to get sicker."

**check pall care referral, see Item #2

Item 2: **Current goals for care.**

		**Question 5: Patient perceives that healthcare currently received is focused on:**			
		EXTENDING LIFE	QUALITY OF LIFE	NOT SURE	Skip
**Question 4:** At this time, if patient had to choose, he/she prefers to focus on:	EXTENDING LIFE	"From your survey, it sounds like you feel your care is focused on the right goals – focusing on extending life as much as possible. Is that right?"	"Some people prefer care focused on extending life as much as possible; other people prefer care focused on quality of life and comfort. Do you have a sense of what is most important to you?"	"Some people prefer care focused on extending life as much as possible; other people prefer care focused on quality of life and comfort. Would it be helpful for me to clarify how I see the focus of your care?"	"Some people prefer care focused on extending life as much as possible; other people prefer care focused on quality of life and comfort. Would it be helpful for me to clarify how I see the focus of your care?"
	*QUALITY OF LIFE	"Some people prefer care focused on extending life as much as possible; other people prefer care focused on quality of life and comfort. Do you have a sense of what is most important to you?"	"From your survey, it sounds like you feel your care is focused on the right goals – focusing on comfort and quality of life. Is that right?"	"Some people prefer care focused on extending life as much as possible; other people prefer care focused on quality of life and comfort. Would it be helpful for me to clarify how I see the focus of your care?"	"Some people prefer care focused on extending life as much as possible; other people prefer care focused on quality of life and comfort. Would it be helpful for me to clarify how I see the focus of your care?"
	NOT SURE	"Some people prefer care focused on extending life as much as possible; other people prefer care focused on quality of life and comfort. Do you have a sense of what is most important to you?"	"Some people prefer care focused on extending life as much as possible; other people prefer care focused on quality of life and comfort. Do you have a sense of what is most important to you?"	"Some people prefer care focused on extending life as much as possible; other people prefer care focused on quality of life and comfort. Do you have a sense of what is most important to you?"	"Some people prefer care focused on extending life as much as possible; other people prefer care focused on quality of life and comfort. Do you have a sense of what is most important to you?"
	Skip	"Some people prefer care focused on extending life as much as possible; other people prefer care focused on quality of life and comfort. Do you have a sense of what is most important to you?"	"Some people prefer care focused on extending life as much as possible; other people prefer care focused on quality of life and comfort. Do you have a sense of what is most important to you?"	"Some people prefer care focused on extending life as much as possible; other people prefer care focused on quality of life and comfort. Do you have a sense of what is most important to you?"	"Some people prefer care focused on extending life as much as possible; other people prefer care focused on quality of life and comfort. Do you have a sense of what is most important to you?"

PICSI-P clinician-patient Jumpstart Matrix 11-8-18

© 2022 Lee RY et al. *JAMA Network Open.*

Item 3: **<u>Barriers to talking about goals</u>**

<table>
<tr><td>1. I don't know what kind of care I would want if I were to get very sick.</td><td>“Even if you aren’t sure what kind of care you want in the future, it is helpful to me if we talk about the things that are important to you so that we can provide the right care.”</td></tr>
<tr><td>2. I'm not ready to talk about the care I would want if I were to get very sick.</td><td rowspan="2">“Some people find it hard to talk about their healthcare in the future. Would you consider giving it a try for a few minutes? At any time, you can just say, ‘Ok, that’s enough for today.’”</td></tr>
<tr><td>3. I don’t like to talk about getting very sick.</td></tr>
<tr><td>~~My clinician doesn't like to talk about my getting very sick.~~</td><td rowspan="2">“Sometimes people worry that doctors don’t have enough time or wouldn’t agree with your thoughts. I want you to know that I consider talking about this really important.”</td></tr>
<tr><td>4. My doctor never seems to have the time to talk about issues like end-of-life care.</td></tr>
<tr><td>5. I would rather concentrate on staying alive than talk about death.</td><td rowspan="2">“Some people find it hard to talk about their healthcare in the future. Would you consider giving it a try for a few minutes? At any time, you can just say, ‘Ok, that’s enough for today.’”</td></tr>
<tr><td>6. I feel that talking about death can bring death closer.</td></tr>
<tr><td>7. I have a living will, and that means I don't need to talk with my doctor about the care I would want if I were too sick to speak for myself.</td><td>“Even though you said in the survey you have completed a living will, it is helpful for me to make sure we are on the same page about what is important to you about your care.”</td></tr>
<tr><td>8. My ideas about the kind of medical care I want change at different times.</td><td>“This is a big topic. Some people need time before they know what decisions would be best. Others worry that they might change their mind in the future. Either way, it still helps me to know something about what you think, even if it’s not final.”</td></tr>
<tr><td>9. I have <u>not</u> felt sick enough to talk with my doctor about end-of-life care.</td><td>“Even though you are doing pretty well, it is helpful for me if I can check my understanding about what is most important to you and the type of care that you want.”</td></tr>
<tr><td>10. I’m not sure which doctor would be taking care of me if I were to get very sick.</td><td>“Although you see a lot of doctors and it is hard to know which of us will be there if you get very sick, it is still helpful for us to talk about it and I can pass on any thoughts you have on the topic to other doctors.”</td></tr>
</table>

** If there is no single “biggest / most important” barrier endorsed, we will feedback one barrier, chosen at random, of those that were endorsed.

** If no barriers are endorsed, then facilitators will be included.

** If no barriers OR facilitators are endorsed then use… “You didn’t mention anything on your survey that makes these discussions easier or harder. Anything come to mind right now?”

Item 3{alt}: **<u>Facilitators to talking about goals</u>**

1. I <u>have</u> been very sick so it is easier to talk about.	“You mentioned on the survey that you have some personal experience with illness. Could you tell me about how your experience influences your thinking?”
2. I have had family or friends who have died so it is easier to talk about.	“You mentioned on the survey that you have some personal experience with illness in your family. Could you tell me about how your experience influences your thinking?”
3. I worry about the quality of my life in the future.	“You mentioned on the survey that you worry about your quality of life in the future. It would be helpful for me if I understood what you consider to be important for your quality of life.”
4. I worry that I could be a burden on my friends and family if I were to become very sick.	“You mentioned on the survey that you worry about being a burden on your family or friends. It can reduce the burden on them if you talk with me and with them about the kind of care you would want if you were to get very sick.”

** If there is no “biggest / most important” facilitator endorsed, we will feedback one facilitator, chosen at random, of those that were endorsed.

** If no barriers OR facilitators are endorsed then use, “You didn’t mention anything on your survey that makes these discussions easier or harder. Anything come to mind right now?”

PICSI-P clinician-patient Jumpstart Matrix 11-8-18

© 2022 Lee RY et al. *JAMA Network Open.*

Item 4: **Preferences for CPR**

		b. In state of dependence on others for ADLs [confined to bed]		
		DEFINITELY or PROBABLY WANTS CPR	DEFINITELY or PROBABLY DOES NOT WANT CPR	Skip
a. **In current state of health**	DEFINITELY or PROBABLY WANTS CPR	"You indicated you would want to receive CPR if your heart were to stop beating. Is that correct? Are there treatments or situations you would not want?"	"You indicated you would want to receive CPR if your heart were to stop beating in your current health, but not if you were to get much sicker and be dependent on others. Is that correct?"	"You indicated you would want to receive CPR if your heart were to stop beating. Is that correct? Are there treatments or situations you would not want?"
	DEFINITELY or PROBABLY DOES NOT WANT CPR	"You indicated you would not want to receive CPR if your heart were to stop beating in your current health, but you would want it if you were much sicker and dependent on others. Is that correct? Can you tell me more about that?"	"You indicated you would not want to receive CPR if your heart were to stop beating. Is that correct?" If yes, consider completing a DNAR order now and a POLST form prior to discharge or a Palliative Care consult.	"You indicated you would not want to receive CPR if your heart were to stop beating. Is that correct?" If yes, consider completing a DNAR order now and a POLST form prior to discharge or a Palliative Care consult.
	Skip	"You indicated you would want to receive CPR if your heart were to stop beating if you were much sicker and dependent on others. Is that correct? Can you tell me more about your CPR preferences in your current health?"	"You indicated you would not want to receive CPR if you were much sicker and dependent on others. Is that correct? Can you tell me more about your CPR preferences in your current health?"	**Choose either text based on question 4**<br>**Text for comfort**<br>"Since you said you would be interested in care focused on comfort and quality of life, let's talk about whether CPR would help you achieve those goals."<br><br>Text for longevity:<br>"Since you said you would be interested in longevity, let's talk about whether CPR would help you achieve those goals."

** If there is no information for this section, e.g. patient skipped this page, then use: "[skipped this page of survey]" or "[skipped this item]" and recommend:

TEXT if goals of care are skipped AND CPR preferences are skipped or if goals of care answer is "I don't know":
"If you were to die unexpectedly, would you want us to use CPR to try to bring you back?

© 2022 Lee RY et al. *JAMA Network Open.*

eAppendix 2. Supplemental Methods

Chart abstraction methods

To measure the primary outcome, we trained a team of four experienced research coordinators in our codebook and abstraction protocol and assigned a subset of participants to each abstractor for primary abstraction. Abstractors used a cloud-based data analysis platform to perform primary abstraction of all clinical notes written during the index hospitalization, and the abstraction team met twice weekly with investigators and a senior research coordinator to review coded excerpts and results. To ensure reliability, at least 5% of each abstractor's allotted charts were co-abstracted by a senior research coordinator. All instances of borderline cases or disagreement were resolved by consensus. The abstractors' guide and codebook are presented in **eAppendix 3**.

Aggregation of abstraction results to primary and comparison outcomes

Following completion of data abstraction, we then determined the primary trial outcome for each patient using the union of chart abstraction results for all clinical notes written between the dates of randomization and discharge. Additionally, to allow comparison of the EHR-documented outcome to patient or surrogate follow-up questionnaire results (discussed below), we also determined a comparison outcome defined by the union of chart abstraction results over the same time frame as the follow-up questionnaire (i.e. from admission to follow-up questionnaire).

Other outcomes

Patient- or surrogate-reported goals-of-care discussions: Patients' or their surrogates' perceptions of having had a goals-of care discussion was assessed with a single question, "During this hospital stay, have you discussed with any of the doctors, in a face-to-face discussion, the kind of medical care you would want if you were too sick to speak for yourself?" Response options included: "yes", "no", "I don't know".

Patient- or surrogate-reported quality of communication: Patient- or surrogate-reported quality of communication was measured using the 7-item end-of-life subscale of the Quality of Communication (QOC) survey.[101-104] In each item, respondents are asked to rate the quality of clinician communication for a given content domain using an 11-point ordinal scale (responses from 0 to 10). For each item, respondents are also able to indicate if the doctor did not address the content domain ("The doctor has not done this.") or if they are unable to provide a rating ("I do not know").

Acceptability of the Jumpstart Guide: Patients or their surrogates and clinicians who participated in the intervention arm completed investigator-developed questions to evaluate the acceptability of the Jumpstart Guide. Acceptability included items assessing use (i.e., "Did you use the Jumpstart Guide?"), usefulness (e.g., "Did the Jumpstart Guide help you start a conversation with your doctor/patient?") and willingness to recommend the intervention (i.e., "Would you recommend the Jumpstart Guide to other patients/clinicians?"). Patients or their surrogates completed five

© 2022 Lee RY et al. *JAMA Network Open.*

questionnaire items assessing acceptability, and clinicians completed three items assessing acceptability.

Feasibility: Feasibility of implementation was assessed through the percentages of patient or surrogate and clinician participants who received Jumpstart Guides.

Patient and clinician questionnaires are presented in **eAppendix 1**.

Participant incentives

Participating patients and surrogates received a $5 token of appreciation for returning each of the baseline and follow-up questionnaires. Clinicians received $5 coffee cards.

Statistical analysis of Quality of Communication questionnaire results

The effect of the intervention on patient- or surrogate-reported quality of communication was evaluated through latent variable analysis of patient and surrogate Quality of Communication (QOC) questionnaire item responses.[101] We chose to use latent variable analysis to mitigate potential bias arising from the internal structure of the questionnaire, as the QOC questionnaire has not been previously validated as a summed score.[105,106] To evaluate patient and surrogate QOC questionnaire responses, we first examined the unidimensionality of a hypothesized latent quality-of-communication variable measured with all 7 QOC questionnaire items, which were modeled as ordered categorical variables.[107,108] Item responses were recoded into three categories: 0 = the indicated aspect of communication did not occur; 1 = it occurred, and was rated ≤ 9 on a 0-10 scale for quality; and, 2 = it occurred, and was rated 10 on the same scale. We fit a two-group model, estimated with weighted least squares, with unstandardized loadings and thresholds constrained to equality between the control and intervention groups.[109] The extent of misfit between the hypothesized model and the observed data was evaluated with the chi-squared test of fit, requiring a p-value ≥ 0.05 for a judgment of "acceptable fit."[110,111] We then used modification indices to revise the initial measurement model, with the goal of identifying a model with acceptable fit to the observed data. Upon identifying a model with good fit, we compared the control and intervention groups' estimated means for the posited construct to assess differences in patient- or surrogate-reported quality of communication between the control and intervention groups.

To further explore the effect of the intervention on quality of communication, we also compared individual questionnaire item responses between groups using a nonparametric equality-of-medians test, as well as summed quality ratings from all 7 QOC questionnaire items between groups using an independent two-sample *t*-test.

© 2022 Lee RY et al. *JAMA Network Open.*

eAppendix 3. Chart Abstractors' Guide and Codebook

Passages in clinical notes that were coded by chart abstractors with any of the following codes were considered to contain a documented goals-of-care discussion. The full codebook, which includes additional abstractor instructions and additional codes not considered to reflect goals-of-care documentation, is published online.[112]

GOCD: Documentation of *new* goals-of-care discussion

DEFINITION: GOC discussions talk about "the overarching aims of medical care for a patient" for *when the patient becomes sicker or when they are dying.* This includes, but is not limited to, planning for future hospitalizations, worsening of disease, or end-of-life care.

A GOC discussion may be "informed by patients' underlying values and priorities," and may ultimately lead to or include "decisions about the use of, or limitation(s) on, specific medical interventions."[113]

★ Please tag the entirety of the discussion.

→ GOCD sub-codes

Within passages coded as GOCD, please use these sub-codes to tag any clear instances of the following three domains: understanding of illness, treatment preferences , and values/goals.

GOCD-Understanding: Documentation of **preexisting understanding/perspective re: illness**

"What have doctors told you thus far?" This code aims to measure documentation of discussion about the patient or family's global understanding of and/or perspectives on the patient's illness. Assessing this understanding at the beginning of a GOC discussion helps align everyone.
Hint: This type of understanding is usually assessed near the beginning of a GOC discussion.

Examples:

- "Mr. ___'s family understands that he is critically ill and at high risk of dying. They understand that his heart is no longer functioning well enough to support other organs."
- "Ms. ___ has been ill for a long time with worsening CHF and has long felt that this would be the disease that would lead to her death."
- "We discussed the patient's understanding of her illness and her desire to know the results of testing." (Documentation of discussion counts, even though the patient's understanding is not explicitly documented.)
- [I asked her what she heard about what is going on.] "She told me that she heard that she has problems with blood flow in her intestines and that it is causing her pain."

© 2022 Lee RY et al. *JAMA Network Open.*

Do not use for standalone documentation of understanding of treatment options or risks/benefits thereof. Understanding of options/risks/benefits (*"What did I just tell you, just now?"*) is a different assessment that often happens near the end of a discussion; it is a poor marker of quality for a GOC discussion, and is often documented for legal assurances.

Negative examples, if occurring in isolation:

- "Patient and family expressed understanding. All questions answered." (Understanding of what?)
- "Family understands he will die without intubation." (This is not an understanding of illness, but rather an understanding of the consequence of death without intubation.)

GOCD-Values/Goals: Discussions about **values and life goals**

Discussions with patients or surrogates about **what they think is most important in life**, or about **specific life results (goals) desired** by patients/surrogates. Patients might prioritize values such as life extension, independence, cognitive capacity, or comfort. They might express life goals of returning home, or living to see a child get married.

Examples:

- "Ms. ___ said that interacting with her family and her husband ___ are priorities. She does not want to suffer, but also prioritizes longevity enough to be willing to give up some comfort."
- "I talked with patient about his values regarding end-of-life care. He believes that life is worth living as long as his heart is beating."
- "Patient prioritizes his quality of life over quantity."
- "Mr. ___ told us that he wants to be able to leave the hospital and go back to his farm."
- "Family hopes for patient to live long enough so that relative from Europe may visit next month."

GOCD-Treatments: Discussions about **treatment preferences or limitations**

Discussions with patients/surrogates about **specific medical treatments**, such as hospitalization, intensive care, life-sustaining treatments, mechanical ventilation, CPR, dialysis, surgery, etc. May include preferences to receive, or to not receive, specific types of treatments.

Examples:

- "Patient desires no CPR, intubation or artificial nutrition."
- "After discussion of resuscitation outcomes, the patient decided to make herself DNR/DNI."
- "At this stage, the patient would like aggressive treatment for illnesses, including a trial of mechanical ventilation. She is ambivalent about CPR vs DNR."
- "Mr. ___ has expressed his wish to continue chemotherapy, despite a high risk of potential complications."

* **Re: the word "comfort":** The word "comfort" may sometimes refer to the treatment strategy of "comfort care" or "comfort measures only." However, if the text is not reflecting an actual, new transition to a comfort-measures-only treatment strategy (which would be coded NEW-COMFORT), you should always code it as GOCD-Values/Goals.

© 2022 Lee RY et al. *JAMA Network Open.*

Sometimes, treatment preferences and values/goals may overlap, or occur in the same sentence.

Examples:

- "The patient remains very interested in any treatments that may prolong his life." [Can apply both subcodes to this quote, which reflects a preference for life-sustaining treatments and a value of life extension.]
- "Patient states that he would want to be kept alive using artificial measures as long as he is not disabled in the long term." [Can apply both subcodes to this quote, which reflects a preference for life-sustaining treatments and a value/goal of avoiding disability.]
- "Patient stated today that she would not want aggressive interventions if she were not able to interact with her family." [Can apply both subcodes to this quote, which reflects a preference for avoiding aggressive interventions contingent upon outcomes conflicting with the value of being able to interact with family.]
- "Patient stated that her goals were to be able to recognize and communicate with her family and that she would be okay with living in a nursing home needing help with ADLs for a short term, but not indefinitely." [Can apply code GOCD-Values/Goals to green portion, and GOCD-Treatments to yellow portion.]

Things that are NOT a new GOC discussion:

- Citation of past GOC discussions without a new discussion [Use code CITATION.]
- Citation of past ACP documents without revision [Use code CITATION.]
- Discussions that do not pertain to *overarching aims* for *when the patient is sicker or dying:*
  - "Goal of physical therapy is for patient to be able to walk up 4 stairs prior to discharge."
  - "Patient wishes to take fewer medicines and reduce pill burden. Will stop atorvastatin."
  - "Patient wants to go to Europe but is afraid of flying. Will prescribe propranolol."
- Shared decision-making about routine medical care:
  - "I discussed with pt whether to pursue prostate ca screening. After deliberation, he declined."
  - "We reviewed the risks and benefits of monitoring this lung nodule versus biopsying it. The patient and I agreed to defer biopsy for now and check a follow-up CT in 3 months."
- Routine documentation of consent prior to a surgery or procedure:
  - "The risks/benefits/alternatives to the procedure were reviewed; patient consented to surgery."

Notes:

- Subcodes of GOCD should be reserved for instances when the defined concept "jumps out at you with clarity." Some GOC discussions may not contain any such passages.
- Subcodes of GOCD need only be applied within passages that are coded GOCD. Content representing a subcode that occurs outside of a GOC discussion need not be coded.

© 2022 Lee RY et al. *JAMA Network Open.*

NEW-ACP: New or revised ACP document, or conversation re: new/revised ACP doc

Documentation that a **new** or revised advance care planning document was discussed with patient/surrogate, completed, or revised. Advance care planning documents/forms include: advance directives (AD), healthcare directives, healthcare DPOA forms, living wills for healthcare (LW, LWHC), POLST, and others. Examples:

- "Completed POLST today."
- "Completed POLST with patient. He would not want CPR, but would want limited interventions."
- "POLST form given to patient, will bring back next visit."
- "POLST from 2 years ago states comfort-measures only; son confirmed this."
- "Patient and wife recently completed Living Will today and brought it in. We discussed patient preferences if his illness should progress."
- "Patient was given a Five Wishes form which we discussed today and I encouraged her to bring this back at our next visit."

If a decision-maker (DPOA) is being changed or reaffirmed, please apply code NEW-ACP, regardless of whether the paperwork formalizing this change/reaffirmation has been completed. Example:

- "Patient still wants his children to be his decision-makers, as he thinks their relationship is improving." [This is discussion that would lead to a revised healthcare DPOA designation.]

NEW-COMFORT: New transition to comfort care, or conversation re: new transition

Documented new transition to comfort measures only (comfort care).

- "Patient transitioning to comfort measures only."
- "I recommended family consider transitioning to comfort care given poor neurological prognosis."

NEW-HOSPICE: New referral to hospice, or conversation re: new referral

Documented new referral to hospice program or hospice facility.

OR

Documented discussion about new referral to hospice with patient/surrogate. Examples:

- "I recommended patient and husband consider hospice today as she is no longer responding to treatment. They will discuss this over and follow-up next week."
- "Patient prefers to die at home, I suggested hospice may be helpful."
- "Patient wishes to stop chemotherapy and asked about hospice."
- "I will have SW place a referral to Zen Hospice today."

Negative examples that reflect neither a new referral nor discussion surrounding one:

- "Patient may be appropriate for hospice given his end-stage disease." [Use code CONGRESS.]
- "94yoM currently on home hospice who was brought to the ED with …" [Do not code. This documentation does not reflect a new referral, as the patient is already enrolled in hospice.]

© 2022 Lee RY et al. *JAMA Network Open.*

- “The patient had previously been on hospice, but was discharged from hospice due to…” [Do not code. This documentation does not reflect a new referral.]

NEW-PALLIATIVE: New referral to palliative care, or conversation re: new referral

Documented new referral or consultation to palliative care.

OR

Documented discussion about new referral or consultation to palliative care with patient/surrogate. Examples:

- “Palliative aware of patient.” or “Palliative care to see.” or “PC consult in AM.” [Implies placement of referral.]
- “We recommended consulting the Palliative Care Service for help with these issues, and we broached this option with the patient.”
- “I suggested that talking with a palliative care specialist may help ease his anxiety.”
- “Explained to patient what palliative care is and how they might help.”

Negative examples that reflect neither a new referral nor discussion surrounding one:

- “Consider Palliative Care referral if pain is not managed.” [Use code CONGRESS.]

Do not use this annotation for notes written *by* the palliative care service. (Apart from this code, palliative care service notes should be annotated in the same way as any other note.)

eTable 1. Timing of Enrollment and Randomization, Patient or Surrogate Follow-up Questionnaire, and Length of Hospital Stay

Time interval	Days, median (IQR)		
	Total sample	Control group	Intervention group
Hospital admission to enrollment and randomization [a]	4 (2-6)	3 (2-6)	4 (2-7)
Randomization to follow-up questionnaire	3 (2-7)	3 (2-7.25)	3 (2-7)
Randomization to hospital discharge	4 (2-9)	4 (2-8)	4 (2-9)
Hospital admission to discharge (i.e., total length of stay)	9 (5-15)	8 (5-14)	9 (6-17)

Abbreviations: IQR, interquartile range

[a] Upon consenting to enrollment, participants were immediately randomized and administered baseline questionnaires.

© 2022 Lee RY et al. *JAMA Network Open.*

eTable 2. Cumulative Incidence of Individual Documentation Domains of EHR-Documented Goals-of-Care Discussions Between Randomization and Discharge

EHR documentation outcome	n (%)	
	Control group *n=75*	Intervention group *n=75*
Goals-of-care discussion (primary outcome; composite of five documentation domains)	6 (8)	16 (21)
Individual documentation domains: [a]		
Discussion of overarching aims of medical care—values, goals, or specific treatment preferences	5 (7)	13 (17)
Discussion of new or revised advance care planning document (e.g. advance directive, living will, POLST, or healthcare DPOA)	2 (3)	3 (4)
Discussion of, or transition to, comfort-measures-only care	3 (4)	5 (7)
Discussion of, or referral to, hospice	1 (1)	6 (8)
Discussion of, or referral to, specialty palliative care for goals of care	3 (4)	7 (9)

Abbreviations: EHR, electronic health record; POLST, Physician Orders for Life-Sustaining Treatment; DPOA, durable power of attorney.

[a] Individual documentation domains are not mutually exclusive.

© 2022 Lee RY et al. *JAMA Network Open.*

eTable 3. Patient- or Surrogate-Reported vs. EHR-Documented Goals-of-Care Discussions Between Dates of Hospital Admission and Follow-up Survey

		EHR-documented goals-of-care discussions between dates of hospital admission and follow-up survey, n (% of column) [a]		
		Yes	No	**TOTAL**
Patient- or surrogate-reported goals-of-care discussions since hospital admission, at follow-up survey	Yes	15 (56)	51 (41)	66 (44)
	No	8 (30)	54 (44)	62 (41)
	Don't know	0 (0)	4 (3)	4 (3)
	Missing [b]	4 (15)	14 (11)	18 (12)
	**TOTAL**	27	123	150

Abbreviations: EHR, electronic health record.
[a] In contrast to the primary trial outcome, this comparator outcome also includes EHR-documented goals-of-care discussions that occurred during the index hospitalization *prior to* randomization, and excludes EHR-documented goals-of-care discussions that occurred after the follow-up survey. This time-frame matches the wording of the patient and surrogate follow-up questionnaires.
[b] There were 18 respondents who did not return a follow-up questionnaire (n=16) or skipped this question (n=2).

© 2022 Lee RY et al. *JAMA Network Open.*

eTable 4. Patient- or Surrogate-Reported Occurrence of a Goals-of-Care Discussion Since Hospital Admission, at Time of Baseline and Follow-up Questionnaires

		Baseline questionnaire, n (% of column)			
		Yes	No	Unsure	**TOTAL**
Follow-up questionnaire	Yes	37 (59)	26 (33)	3 (33)	66 (44)
	No	18 (29)	39 (50)	5 (56)	62 (41)
	Don’t know	2 (3)	1 (1)	1 (11)	4 (3)
	Missing [a]	6 (10)	12 (15)	0 (0)	18 (12)
	**TOTAL**	63	78	9	150

[a] There were 18 respondents who answered this question in the baseline questionnaire but did not provide an answer at follow-up (16 who did not complete a follow-up questionnaire and 2 who skipped this question on the follow-up questionnaire).

© 2022 Lee RY et al. *JAMA Network Open.*

eFigure 3. Two-Group Five-Indicator Latent Quality-of-Communication Model

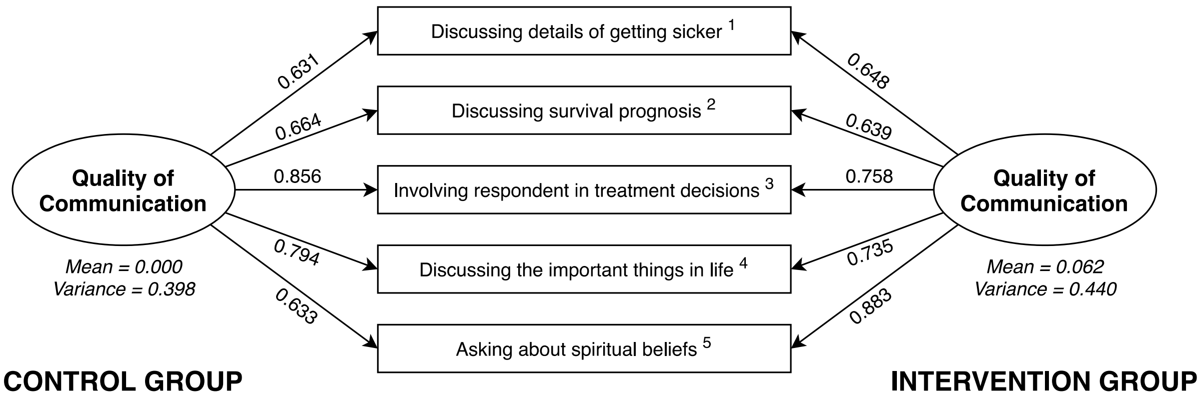

CONTROL GROUP

Estimated R^2

1. *0.398*
2. *0.441*
3. *0.733*
4. *0.631*
5. *0.401*

Unstandardized

	loadings	***thresholds***
1.	*1.000*	*-0.319, 0.831*
2.	*1.052*	*0.724, 1.402*
3.	*1.358*	*-0.059, 0.982*
4.	*1.259*	*0.010, 1.238*
5.	*1.003*	*0.392, 0.993*

(constrained to equality between control and intervention groups)

INTERVENTION GROUP

Estimated R^2

1. *0.419*
2. *0.409*
3. *0.574*
4. *0.540*
5. *0.780*

Test of fit: χ^2 = 19.957, 18 df, p = 0.3352
Comparative fit index (CFI) = 0.993
Tucker-Lewis index (TLI) = 0.993

© 2022 Lee RY et al. *JAMA Network Open.*

eTable 5. Effect of Intervention on Patient or Surrogate Quality of Communication Questionnaire Responses at Follow-up

Outcome	Total sample N = 134/150 [a]		Control group N = 66/75 [a]		Intervention group N = 68/75 [a]		*p* [c]
	N [b]	n (%)	N [b]	n (%)	N [b]	n (%)	
Discussing feelings about getting sicker	122		61		61		0.81
Not addressed		47 (39)		25 (41)		22 (36)	
Addressed, quality rating ≤ 9		53 (43)		25 (41)		28 (46)	
Addressed, quality rating 10		22 (18)		11 (18)		11 (18)	
Discussing details of getting sicker	126		65		61		0.85
Not addressed		46 (37)		22 (34)		24 (39)	
Addressed, quality rating ≤ 9		53 (42)		30 (46)		23 (38)	
Addressed, quality rating 10		27 (21)		13 (20)		14 (23)	
Discussing survival prognosis	126		65		61		0.41
Not addressed		94 (75)		51 (78)		43 (70)	
Addressed, quality rating ≤ 9		20 (16)		9 (14)		11 (18)	
Addressed, quality rating 10		12 (10)		5 (8)		7 (11)	
Discussing what dying might be like	130		66		64		0.41
Not addressed		112 (86)		59 (89)		53 (83)	
Addressed, quality rating ≤ 9		11 (8)		4 (6)		7 (11)	
Addressed, quality rating 10		7 (5)		3 (5)		4 (6)	
Involving respondent in treatment decisions	125		64		61		0.72
Not addressed		58 (46)		31 (48)		27 (44)	
Addressed, quality rating ≤ 9		43 (34)		22 (34)		21 (34)	
Addressed, quality rating 10		24 (19)		11 (17)		13 (21)	
Discussing the important things in life	125		62		63		0.82
Not addressed		61 (49)		33 (53)		28 (44)	
Addressed, quality rating ≤ 9		48 (38)		21 (34)		27 (43)	
Addressed, quality rating 10		16 (13)		8 (13)		8 (13)	
Asking about spiritual beliefs	127		64		63		0.95
Not addressed		84 (66)		42 (66)		42 (67)	
Addressed, quality rating ≤ 9		26 (20)		12 (19)		14 (22)	
Addressed, quality rating 10		17 (13)		10 (16)		7 (11)	
Quality of Communication end-of-life subscale composite score, mean (standard deviation) [d]	101	26.1 (21.5)	52	23.9 (20.2)	49	28.5 (22.8)	0.28

[a] Number of patient or surrogate follow-up questionnaire respondents, over number of participating patients or surrogates.
[b] For individual questionnaire items, number of respondents who provided a quality rating for discussion of the content domain or reported that it was not addressed; "I do not know" was treated as missing. For composite score, number of respondents who provided non-missing responses to all required items.
[c] For individual items, *p*-value by 2-sided median test of ordinal categories shown. For composite score, *p*-value by independent two-sample *t*-test.

© 2022 Lee RY et al. *JAMA Network Open.*

[d] Composite score was computed as the sum of all 7 items (coded as 0: not addressed; 1-11: original 0-10 quality rating) for respondents with non-missing responses to all 7 required items.

© 2022 Lee RY et al. *JAMA Network Open.*

eReferences

101. Engelberg R, Downey L, Curtis JR. Psychometric characteristics of a quality of communication questionnaire assessing communication about end-of-life care. *J Palliat Med.* 2006;9(5):1086-1098.
102. Curtis JR, Engelberg RA, Nielsen EL, et al. Patient-physician communication about end-of-life care for patients with severe COPD. *Eur Respir J.* 2004;24(2):200-205.
103. Curtis JR, Patrick DL, Caldwell E, et al. The quality of patient-doctor communication about end-of-life care: a study of patients with advanced AIDS and their primary care clinicians. *AIDS.* 1999;13(9):1123-1131.
104. Au DH, Udris EM, Engelberg RA, et al. A randomized trial to improve communication about end-of-life care among patients with COPD. *Chest.* 2012;141(3):726-735.
105. Cook DA, Beckman TJ. Current concepts in validity and reliability for psychometric instruments: theory and application. *Am J Med.* 2006;119(2):166 e167-116.
106. McNeish D, Wolf MG. Thinking twice about sum scores. *Behav Res Methods.* 2020;52(6):2287-2305.
107. Bollen KA, Hoyle RH. Latent variables in structural equation modeling. In: Hoyle RH, ed. *Handbook of Structural Equation Modeling.* New York: Guilford Press; 2012:56-57.
108. Bovaird JA, Koziol NA. Measurement models for ordered-categorical indicators. In: Hoyle RH, ed. *Handbook of Structural Equation Modeling.* New York: Guilford Press; 2012:495-511.
109. Millsap RE, Olivera-Aguilar M. Investigating measurement invariance using confirmatory factor analysis. In: Hoyle RH, ed. *Handbook of Structural Equation Modeling.* New York: Guilford Press; 2012:380-392.
110. Hayduk LA. *LISREL Issues, Debates, and Strategies.* Baltimore; 1996.
111. West SG, Taylor AB, Wu W. Model fit and model selection in structural equation modeling. In: Hoyle RH, ed. *Handbook of Structural Equation Modeling.* New York: Guilford Press; 2012:209-231.
112. Lee RY, Okimoto K, Treece PD, et al. Chart Abstractor Codebook for Project to Improve Communication in Serious Illness - Pilot Study (PICSI-P). 2020; https://faculty.washington.edu/rlee06/picsi-p-public/PICSI-P-Abstractor-Codebook.pdf. Accessed Jan 29, 2021.
113. Secunda K, Wirpsa MJ, Neely KJ, et al. Use and Meaning of "Goals of Care" in the Healthcare Literature: a Systematic Review and Qualitative Discourse Analysis. *J Gen Intern Med.* 2019;35(5):1559-1566.
114. Harris PA, Taylor R, Thielke R, et al. Research electronic data capture (REDCap)--a metadata-driven methodology and workflow process for providing translational research informatics support. *J Biomed Inform.* 2009;42(2):377-381.

© 2022 Lee RY et al. *JAMA Network Open.*
