# Supplementary material for: Efficacy of a Communication-Priming Intervention on Documented Goals-of-Care Discussions in Hospitalized Patients With Serious Illness: A Randomized Clinical Trial
Source: JAMA Netw Open. 2022 Apr 1;5(4):e225088. doi: 10.1001/jamanetworkopen.2022.5088 (PMC8976242; doi:10.1001/jamanetworkopen.2022.5088)
Supplement: Supplement 3. — Data Sharing Statement [file jamanetwopen-e225088-s003.pdf]

Data Sharing Statement

Lee. Efficacy of a Communication-Priming Intervention on Documented Goals-of-Care Discussions in Hospitalized Patients With Serious Illness. *JAMA Netw Open*. Published April 01, 2022. doi:10.1001/jamanetworkopen.2022.5088

Data
Data available: No

Additional Information
Explanation for why data not available: Pilot trial without opportunity for significant secondary data analyses
